# Knowledge, attitudes and practices on childhood TB among healthcare workers

**DOI:** 10.5588/ijtld.21.0317

**Published:** 2022-03-01

**Authors:** B. Joshi, H. Font, E. Wobudeya, M. Nanfuka, A. Kobusingye, J. Mwanga-Amumpaire, N. Natukunda, S. Turyahabwe, L. Borand, T. E. Mao, B. Dim, R. Ferhi, R. Moh, J. Kouakou, R. Aka Bony, G. Breton, A. Mustapha, L. Matata, L. Foray, A. Detjen, S. Verkuijl, M. Sekadde, C. Khosa, V. Mbassa, J-V. Taguebue, S. Kwedi Nolna, M. Bonnet, O. Marcy, J. Orne-Gliemann

**Affiliations:** 1 Unité mixte de recherche 1219, University of Bordeaux, Institut national de la santé et de la recherche médicale (INSERM), Institut de Recherche pour le Développement (IRD) EMR 271, Bordeaux Population Health Centre, Bordeaux, France; 2 Makerere University–Johns Hopkins University (MU-JHU) Research Collaboration, MU-JHU Care Limited, Kampala, Uganda; 3 Epicentre Mbarara Research Centre, Mbarara, Uganda; 4 National Tuberculosis and Leprosy Program, Kampala, Uganda; 5 Epidemiology and Public Health Unit, Institut Pasteur du Cambodge, Phnom Penh, Cambodia; 6 Centre national de Lutte contre la Tuberculose et la Lèpre (CENAT), Phnom Penh, Cambodia; 7 Programme ANRS Coopération Côte d’Ivoire (PAC-CI) Abidjan, Côte d’Ivoire; 8 Programme National de Lutte contre la Tuberculose (PNLT), Abidjan, Côte d’Ivoire; 9 Solthis, Paris, France; 10 Ola During Children’s Hospital, Freetown, Sierra Leone; 11 Solthis, Freetown, Sierra Leone; 12 National Leprosy and TB Control Programme, Freetown, Sierra Leone; 13 Child and Community Health Unit, United Nations Children’s Fund (UNICEF), New York, NY, USA; 14 Global Tuberculosis Programme, WHO, Geneva, Switzerland; 15 Instituto Nacional de Saúde, Maputo, Mozambique; 16 PNLT, Yaoundé, Cameroon; 17 Mother and Child Centre, Chantal Biya Foundation, Yaoundé, Cameroon; 18 IRD, Yaoundé, Cameroon; 19 TransVIHMI (Recherches Translationnelles sur le VIH et les Maladies Infectieuses), University of Montpellier, IRD, INSERM, Montpellier, France

**Keywords:** knowledge, childhood TB, tuberculosis, healthcare workers, LMICs

## Abstract

**BACKGROUND:**

Increasing childhood TB case detection requires the deployment of diagnostic services at peripheral healthcare level. Capacity and readiness of healthcare workers (HCWs) are key to the delivery of innovative approaches.

**METHODS:**

In 2019, HCWs from five district hospitals (DHs) and 20 primary healthcare centres (PHCs) in Cambodia, Cameroon, Cote d’Ivoire, Sierra Leone and Uganda completed a self-administered knowledge-attitudes-practices (KAP) questionnaire on childhood TB. We computed knowledge and attitudes as scores and identified HCW characteristics associated with knowledge scores using linear regression.

**RESULT:**

Of 636 eligible HCWs, 497 (78%) participated. Median knowledge scores per country ranged between 7.4 and 12.1 (/18). Median attitude scores ranged between 2.8 and 3.3 (/4). Between 13.3% and 34.4% of HCWs reported diagnosing childhood with (presumptive) TB few times a week. Practising at PHC level, being female, being involved in indirect TB care, having a non-permanent position, having no previous research experience and working in Cambodia, Cameroon, Cote d’Ivoire and Sierra Leone as compared to Uganda were associated with a lower knowledge score.

**CONCLUSION:**

HCWs had overall limited knowledge, favourable attitudes and little practice of childhood TB diagnosis. Increasing HCW awareness, capacity and skills, and improving access to effective diagnosis are urgently needed.

## Abstract (fr)

**CONTEXTE:**

L’augmentation du dépistage des cas de TB infantile nécessite le déploiement des services de diagnostic au niveau des soins de santé périphériques. Les compétences et la motivation des agents de santé sont essentielles à la mise en place d’approches innovantes.

**MÉTHODES:**

En 2019, les agents de santé de cinq hôpitaux de district (DH) et de 20 centres de soins de santé primaires (PHC) au Cambodge, au Cameroun, en Côte d’Ivoire, en Sierra Leone et en Ouganda ont rempli un questionnaire auto-administré sur leurs connaissances, attitudes et pratiques (KAP) en matière de TB infantile. Nous avons calculé des scores de connaissance et d’attitude et identifié les caractéristiques des agents de santé associées aux scores de connaissance à l’aide d’une régression linéaire.

**RÉSULTATS:**

Sur 636 HCW éligibles, 497 (78%) ont participé. Les scores médians de connaissance par pays variaient entre 7,4 et 12,1 (/18). Les scores médians d’attitude variaient entre 2,8 et 3,3 (/4). Entre 13,3% et 34,4% des agents de santé ont déclaré qu’ils diagnostiquaient une TB (présomptive) chez l’enfant plusieurs fois par semaine. Exercer en PHC, être une femme, prendre part aux soins indirects de la TB, avoir un emploi non-permanent, n’avoir aucune expérience antérieure de recherche et travailler au Cambodge, au Cameroun, en Côte d’Ivoire et en Sierra Leone par rapport à l’Ouganda, étaient associés avec un score de connaissance inférieur.

**CONCLUSION:**

Les HCW possédaient des connaissances globales limitées, avaient une attitude favorable et peu d’expérience en matière de diagnostic de la TB infantile. Il est urgent d’améliorer la sensibilisation et les compétences des HCW et d’améliorer l’accès à un diagnostic efficace de la TB chez l’enfant.

Childhood TB remains largely undiagnosed. Of an estimated 1.2 million childhood TB cases in 2019, only 522,000 (44%) were reported to the WHO.^1^ From a patient perspective, challenges to childhood TB diagnosis include 1) the paucibacillary nature of TB in children, contributing to poor sensitivity of microbiological tests; 2) difficulties in obtaining respiratory samples, especially since young children under 5 are unable to self-expectorate; 3) poor specificity of symptoms, in particular, in those with HIV infection or severe acute malnutrition; 4) complicated chest radiography (CXR) features that are difficult to interpret.^2^–^4^ From a health systems perspective, challenges to childhood TB diagnosis include 1) poor availability of diagnostic tools at primary health centre (PHC) level; 2) limited number of healthcare workers (HCWs) in PHCs who are trained and skilled in conducting proper TB screening, clinical evaluation and sample collection, and in referring children with presumptive TB when needed; and 3) poor availability and poor quality of CXR imaging and limited reading skills.^5^–^7^ Poor awareness of childhood TB among community members also poses a challenge to proper management.^8^ All these factors contribute to the overall TB case detection gap among children, which fuels childhood TB mortality.^9^

The WHO recently recommended alternative childhood TB specimens such as stool and nasopharyngeal aspirate (NPA) that may be simpler to collect in resource-limited health settings.^10^ The Xpert^®^ MTB/RIF assay (Xpert; Cepheid, Sunnyvale, CA, USA), which had been recommended by the WHO for childhood TB testing and diagnosis based on cumulative evidence,^11^,^12^ has now been superseded by the newly developed Xpert Ultra (Ultra; Cepheid) test, which has improved sensitivity.^4^,^13^,^14^ Nevertheless, Ultra remains underutilised in children due to challenges in accessing and implementing child-friendly sample collection.^15^,^16^ Digital radiography could be used to solve the problem of inferior CXRs due to poor quality reagents, making it easier to interpret and transfer images for external reading and quality control, or ultimately the use of computer-aided diagnosis after full validation in children.^17^ Decentralisation of child TB services, improved community awareness about childhood TB and strengthened community-based TB screening/linkages can increase case-finding.^18^,^19^

HCWs are key actors in implementing interventions to improve childhood TB diagnosis.^20^ There is evidence of sub-optimal levels knowledge about TB diagnosis, treatment and infection prevention and control, negative attitudes and stigma towards TB, as well as poor care practices among HCWs,^21^,^22^ which may negatively impact patients and communities. Documenting levels of knowledge and experience among HCWs, opinions about current services and perceptions about potential benefits of planned interventions, help assess HCW capacity and preparedness in the delivery of innovative approaches, that play an important role in intervention success.

We sought to assess knowledge gaps on childhood TB among HCWs, their perceptions and attitudes towards TB and TB patients, as well as their level of experience in childhood TB, prior to implementing a large operational research study on childhood TB diagnosis in five high TB incidence countries. We also aimed to identify key sociodemographic and health systems factors associated with levels of knowledge among HCWs.

## METHODS

### Study design and setting

We conducted a knowledge-attitudes-practices (KAP) survey in Cambodia, Cameroon, Côte d’Ivoire (CI), Sierra Leone (SL) and Uganda as part of the TB-Speed Decentralisation Study (ClinicalTrials.gov Identifier: NCT04038632). This study aimed to assess the impact on childhood TB case detection of implementing an innovative diagnostic approach at district hospital (DH) and PHC levels. The diagnostic approach included systematic TB screening using symptoms and contact history of any sick child at triage, NPA and stool sample collection and Ultra testing for microbiological diagnosis, optimised CXR using a digitalised system, simplified reading tools and external quality control, and strengthening of clinical diagnosis skills through training and mentoring. Two rural/semi-rural health districts, with one DH and four PHCs per district, were targeted in each country.

### Study population and sample size

HCWs who were either currently or soon to be (as part of the TB-Speed Decentralisation Study) involved in the management of childhood TB were eligible. We did not pre-define sample size as we aimed for exhaustive recruitment of all those meeting our selection criteria.

### KAP questionnaire

We developed the KAP questionnaire (Supplementary Data 2) based on a literature review of KAP surveys on TB/childhood TB and multidisciplinary insight from national and international TB experts. We pretested the questionnaire among 10 HCWs in a Uganda non-study PHC in June 2019. Knowledge questions focused on epidemiology (transmission, risk), diagnosis (clinical, microbiological), treatment and prevention (vaccine, contact tracing, TB preventive treatment [TPT]). Attitudes towards TB risk, screening, sample collection and treatment were assessed using a 4-point Likert scale. Practices questions focused on screening, diagnosis and treatment of childhood TB activities, prevention/education activities, contact tracing and use of TPT at the facility level. Two additional modules were used to assess satisfaction with local health services and community perceptions of childhood TB.

### Data collection procedures

We conducted the KAP survey between July and September 2019. The health facility in-charge listed all eligible HCWs. HCWs agreeing to participate received detailed information about the survey as a group. After individual consent had been provided, HCWs were gathered in a single room and responded to the self-administered questionnaire on paper or tablet using REDCap (Research Electronic Data Capture mobile application v4.9.1, 6 February 2020; Vanderbilt University, Nashville, TN, USA).

### Data analysis

We summarised knowledge and attitudes as scores. Knowledge questions were attributed points from 0 to 1: 1 point was awarded for the correct answer and 0 for the wrong answer in case of single-correct answer questions; for multiple correct-answer questions, each correct answer was graded 1/number of correct answers, and each wrong answer graded an equivalent negative number, with a minimum score of 0 (Supplementary Data 3). A score per pre-defined knowledge dimensions, i.e., epidemiology, diagnosis, treatment or prevention, was computed by summing the correct answers to the corresponding questions (maximum score of 4 or 5 points) and an overall knowledge score was calculated by summing the scores of the four dimensions (maximum of 18 points). Attitudes were scored using discrete numbers from 1 to 4. A higher attitudes score indicated a more positive attitude towards childhood TB. A score was computed post-hoc for three attitudes dimensions, namely emotional (referring to feelings or emotions), cognitive (referring to beliefs or thoughts) and behavioural (referring to acts or behaviours).^23^ The overall attitude score was the mean of all attitude scores. Practices were described in percentages.

We assessed the association between HCW characteristics and the global knowledge score using linear regression. Variables associated with knowledge at a threshold of 0.25 in univariable analysis were included in the multivariable linear model. We obtained the final model by stepwise backward selection, with α = 0.05. All analyses were performed using the R Software v3.6.1 (R Computing, Vienna, Austria).

### Ethics approval

The KAP survey was implemented as part of the TB-Speed Decentralisation Study, approved by the ethics review committees of WHO, INSERM (Institut national de la Santé et de la Recherche Médicale, Paris, France), and participant countries, and relevant institutional review boards.

## RESULTS

Of the 636 eligible HCWs, a total of 497 (78%) completed the KAP questionnaire. The median age of respondents ranged between 31.0 years in Cameroon and 40.0 years in Cambodia, the proportion of PHC-based HCWs ranged from 43.0% in Sierra Leone to 69.7% in Cameroon, and the proportion of staff involved in direct TB care ranged from 40.0% in Cambodia to 68.7% in Cameroon (Table 1).

**Table 1:** Healthcare workers characteristics

	Cambodia (*n* = 30) *n* (%)	Cameroon (*n* = 99) *n* (%)	Côte d’Ivoire (*n* = 99) *n* (%)	Sierra Leone (*n* = 93) *n* (%)	Uganda (*n* = 176) *n* (%)
Sex					
Male	16 (53.3)	37 (37.4)	59 (59.6)	45 (48.4)	73 (41.5)
Female	14 (46.7)	62 (62.6)	39 (39.4)	48 (51.6)	96 (54.5)
Missing	0 (0.0)	0 (0.0)	1 (1.0)	0 (0.0)	7 (4.0)
Age, years, median [IQR]	40.0 [25.0–62.0]	31.0 [19.9–56.0]	36.1 [18.0–73.6]	36.0 [24.0–63.5]	35.3 [22–63]
Missing	0 (0.0)	0 (0.0)	3 (3.0)	20 (21.5)	1 (0.6)
Position					
Paediatrician	0 (0.0)	0 (0.0)	1 (1.0)	0 (0.0)	0 (0.0)
Clinical officer or medical assistant	0 (0.0)	0 (0.0)	0 (0.0)	21 (22.6)	22 (12.5)
General practitioner	3 (10.0)	15 (15.2)	4 (4.0)	1 (1.1)	8 (4.5)
Nurse	6 (20.0)	24 (24.2)	20 (20.2)	32 (34.4)	60 (34.1)
Nursing assistant	3 (10.0)	29 (29.3)	30 (30.3)	7 (7.5)	3 (1.7)
Midwife	11 (36.7)	4 (4.0)	0 (0.0)	0 (0.0)	21 (11.9)
Laboratory technician or microscopist	1 (3.3)	13 (13.1)	12 (12.1)	20 (21.5)	25 (14.2)
Radiologist	0 (0.0)	0 (0.0)	0 (0.0)	0 (0.0)	0 (0.0)
Radiology technician	2 (6.7)	0 (0.0)	5 (5.1)	4 (4.3)	0 (0.0)
Social assistant or counsellor	0 (0)	1 (1.0)	1 (1.0)	1 (1.1)	10 (5.7)
Community health worker/volunteer	2 (6.7)	7 (7.1)	13 (13.1)	4 (4.3)	11 (6.2)
Other	2 (6.7)	6 (6.1)	12 (12.1)	3 (3.2)	16 (9.1)
Missing	0 (0.0)	0 (0.0)	1 (1.0)	0 (0.0)	0 (0.0)
Position recoded*					
Direct TB care	12 (40.0)	68 (68.7)	56 (56.6)	63 (67.7)	96 (54.5)
Indirect TB care	18 (60.0)	31 (31.3)	43 (43.4)	30 (32.3)	80 (45.5)
Contract type					
Permanent	22 (73.3)	50 (50.5)	41 (41.4)	61 (65.6)	144 (81.8)
Temporary	4 (13.3)	23 (23.2)	8 (8.1)	2 (2.2)	17 (9.7)
Volunteer	0 (0)	20 (20.2)	19 (19.2)	30 (32.3)	12 (6.8)
Other	4 (13.3)	5 (5.1)	15 (15.2)	0 (0.0)	1 (0.6)
Missing	0 (0.0)	1 (1.0)	16 (16.2)	0 (0.0)	1 (0.6)
Research experience					
No	15 (50.0)	64 (64.6)	72 (72.7)	63 (67.7)	107 (60.8)
Yes	11 (36.7)	35 (35.4)	7 (7.1)	17 (18.3)	42 (23.9)
Missing	4 (13.3)	0 (0.0)	20 (20.2)	13 (14.0)	27 (15.3)

* We considered healthcare workers as involved in “direct TB care” if they provided clinical and frontline care, i.e., paediatricians, general practitioners, medical officers or nurses; and involved in “indirect TB care” if they did not provide direct clinical and frontline care, i.e., laboratory technician, radiographers or community health workers.

IQR = interquartile range.

### Knowledge

The overall median knowledge scores ranged between 7.4/18 in Côte d’Ivoire and 12.1/18 in Uganda (Table 2). The highest median scores were obtained for epidemiology. Median diagnosis scores were at or below average (2.5/5) in all countries, except Uganda. More than 75% of the respondents in all countries knew that the causative agent of TB is a bacterium; however, in most countries, less than 50% of HCWs knew that Xpert is a molecular test for TB (Table 3). Most HCWs correctly knew the duration of TB treatment in children. Detailed knowledge results are presented in Supplementary Data 4.

**Table 2:** Global childhood TB knowledge and attitude scores among healthcare workers

	Cambodia (*n* = 30) median [IQR]	Cameroon (*n* = 99) median [IQR]	Côte d’Ivoire (*n* = 99) median [IQR]	Sierra Leone (*n* = 93) median [IQR]	Uganda (*n* = 176) median [IQR]
Knowledge					
Global score (/18)	10.1 [8.03–11.90]	10.2 [8.13–11.80]	7.4 [5.02–9.28]	8.9 [7.07–10.90]	12.1 [10.3–13.80]
Epidemiology (/5)	3.3 [2.75–3.83]	3.8 [3.00–4.33]	3.3 [2.33–3.92]	3.3 [2.83–4.00]	4.2 [3.50–4.50]
Diagnosis (/5)	2.4 [1.55–3.08]	2.2 [1.75–2.95]	1.5 [0.88–1.98]	1.9 [1.35–2.70]	2.9 [2.09–3.35]
Treatment (/4)	2.3 [1.25–2.50]	2.0 [1.25–2.75]	1.0 [0.00–1.75]	2.0 [1.25–2.75]	3.0 [2.25–4.00]
Prevention (/4)	2.0 [1.50–2.88]	2.0 [1.50–2.50]	1.5 [1.00–2.50]	1.5 [1.00–2.50]	2.0 [1.50–3.00]
Attitudes					
Global score (/4)	3.3 [3.13–3.48]	2.8 [2.64–2.98]	2.9 [2.72–3.07]	2.8 [2.64–3.08]	3.1 [2.89–3.29]
Emotional (/4)	2.8 [2.50–3.00]	2.0 [1.75–2.50]	2.5 [2.00–2.75]	2.3 [2.00–2.75]	2.5 [2.00–2.75]
Cognitive (/4)	3.4 [3.26–3.80]	3.0 [2.76–3.29]	3.0 [2.79–3.30]	3.3 [3.00–3.43]	3.4 [3.14–3.71]
Behavioural (/4)	3.8 [3.50–4.00]	3.3 [3.00–3.50]	3.3 [3.00–3.67]	3.0 [2.50–3.25]	3.5 [3.19–4.00]

IQR = interquartile range.

**Table 3:** Selected knowledge, attitudes and practices among healthcare workers

	Cambodia (*n* = 30) *n* (%)	Cameroon (*n* = 99) *n* (%)	Côte d’Ivoire (*n* = 99) *n* (%)	Sierra Leone (*n* = 93) *n* (%)	Uganda (*n* = 176) *n* (%)
Knowledge					
What causes TB?					
A bacteria	23 (76.7)	85 (85.9)	81 (81.8)	79 (84.9)	154 (87.5)
A virus	5 (16.7)	13 (13.1)	10 (10.1)	11 (11.8)	18 (10.2)
A fungus	0 (0.0)	0 (0.0)	0 (0.0)	1 (1.1)	0 (0.0)
Missing	2 (6.7)	1 (1.0)	8 (8.1)	2 (2.2)	4 (2.3)
What is the Xpert^®^ MTB/RIF assay?					
A test for CD4 lymphocytes count	3 (10.0)	11 (11.1)	3 (3.0)	16 (17.2)	14 (8.0)
A type of X-ray to diagnose TB	6 (20.0)	36 (36.4)	33 (33.3)	29 (31.2)	27 (15.3)
A molecular test for TB	15 (50.0)	47 (47.5)	21 (21.2)	36 (38.7)	120 (68.2)
A tuberculin skin test	1 (3.3)	2 (2.0)	8 (8.1)	5 (5.4)	3 (1.7)
Missing	5 (16.7)	3 (3.0)	34 (34.3)	7 (7.5)	12 (6.8)
Total duration of TB treatment in children?					
2 weeks	0 (0.0)	3 (3.0)	4 (4.0)	8 (8.6)	1 (0.6)
2 months	0 (0.0)	10 (10.1)	3 (3.0)	6 (6.5)	6 (3.4)
4 months	0 (0.0)	8 (8.1)	3 (3.0)	8 (8.6)	8 (4.5)
6 months	24 (80.0)	72 (72.7)	59 (59.6)	55 (59.1)	152 (86.4)
12 months	0 (0.0)	6 (6.1)	2 (2.0)	12 (12.9)	4 (2.3)
Missing	6 (20.0)	0 (0.0)	28 (28.3)	4 (4.3)	5 (2.8)
Name of vaccine against TB					
BCG	26 (86.7)	84 (84.8)	52 (52.5)	54 (58.1)	146 (83.0)
Attitudes					
I am reluctant to collect and test stools for TB in children					
Strongly disagree	2 (6.7)	23 (23.2)	10 (10.1)	24 (25.8)	37 (21.0)
Disagree	10 (33.3)	49 (49.5)	38 (38.4)	36 (38.7)	57 (32.4)
Agree	11 (36.7)	15 (15.2)	18 (18.2)	18 (19.4)	52 (29.5)
Strongly agree	6 (20.0)	10 (10.1)	7 (7.1)	14 (15.1)	22 (12.5)
Missing	1 (3.3)	2 (2.0)	26 (26.3)	1 (1.1)	8 (4.5)
I believe any child attending outpatient clinic should be systematically screened for TB					
Strongly disagree	3 (10.0)	7 (7.1)	6 (6.1)	13 (14.0)	7 (4.0)
Disagree	1 (3.3)	35 (35.4)	40 (40.4)	33 (35.5)	30 (17.0)
Agree	6 (20.0)	39 (39.4)	22 (22.2)	24 (25.8)	44 (25.0)
Strongly agree	19 (63.3)	17 (17.2)	16 (16.2)	22 (23.7)	92 (52.3)
Missing	1 (3.3)	1 (1.0)	15 (15.2)	1 (1.1)	3 (1.7)
I believe TB diagnosis in children is more difficult than in adults					
Strongly disagree	0 (0.0)	7 (7.1)	2 (2.0)	4 (4.3)	9 (5.1)
Disagree	1 (3.3)	14 (14.1)	6 (6.1)	6 (6.5)	13 (7.4)
Agree	16 (53.3)	46 (46.5)	45 (45.5)	19 (20.4)	72 (40.9)
Strongly agree	12 (40.0)	32 (32.3)	33 (33.3)	63 (67.7)	80 (45.5)
Missing	1 (3.3)	0 (0.0)	13 (13.1)	1 (1.1)	2 (1.1)
I believe persons with TB signs and symptoms should be screened for TB					
Strongly disagree	0 (0.0)	0 (0.0)	1 (1.0)	2 (2.2)	1 (0.6)
Disagree	0 (0.0)	1 (1.0)	0 (0)	3 (3.2)	0 (0.0)
Agree	3 (10.0)	31 (31.3)	35 (35.4)	16 (17.2)	26 (14.8)
Strongly agree	26 (86.7)	67 (67.7)	59 (59.6)	72 (77.4)	149 (84.7)
Missing	1 (3.3)	0 (0.0)	4 (4.0)	0 (0.0)	0 (0.0)
I believe the majority of staff in health facility have adequate training on childhood TB					
Strongly disagree	3 (10.0)	13 (13.1)	16 (16.2)	27 (29.0)	48 (27.3)
Disagree	14 (46.7)	51 (51.5)	30 (30.3)	33 (35.5)	83 (47.2)
Agree	5 (16.7)	30 (30.3)	21 (21.2)	19 (20.4)	25 (14.2)
Strongly agree	6 (20.0)	5 (5.1)	9 (9.1)	13 (14.0)	18 (10.2)
Missing	2 (6.7)	0 (0.0)	23 (23.2)	1 (1.1)	2 (1.1)
People in my community believe that a child who has persistent cough should be brought to the clinic as soon as possible					
Strongly disagree	1 (3.3)	1 (1.0)	0 (0.0)	4 (4.3)	15 (8.5)
Disagree	8 (26.7)	14 (14.1)	5 (5.1)	12 (12.9)	45 (25.6)
Agree	12 (40.0)	51 (51.5)	41 (41.4)	39 (41.9)	84 (47.7)
Strongly agree	8 (26.7)	32 (32.3)	40 (40.4)	37 (39.8)	31 (17.6)
Missing	1 (3.3)	1 (1.0)	13 (13.1)	1 (1.1)	1 (0.6)
Practices					
How often do you diagnose children with TB or presumptive TB in your health facility?					
Never	8 (26.7)	3 (3.0)	0 (0.0)	9 (9.7)	9 (5.1)
Few times a year	5 (16.7)	26 (26.3)	11 (11.1)	16 (17.2)	51 (29.0)
Few times a month	9 (30.0)	24 (24.2)	17 (17.2)	31 (33.3)	60 (34.1)
Few times a week	4 (13.3)	18 (18.2)	15 (15.2)	32 (34.4)	49 (27.8)
Missing	4 (13.3)	28 (28.3)	56 (56.6)	5 (5.4)	7 (4.0)
To collect sputum for TB diagnosis in a child, do you refer to another centre for sputum collection?					
Never	16 (53.3)	48 (48.5)	26 (26.3)	56 (60.2)	110 (62.5)
Sometimes	4 (13.3)	14 (14.1)	12 (12.1)	19 (20.4)	44 (25.0)
Often	2 (6.7)	11 (11.1)	8 (8.1)	5 (5.4)	4 (2.3)
Always	4 (13.3)	25 (25.3)	7 (7.1)	10 (10.8)	10 (5.7)
Missing	4 (13.3)	1 (1.0)	46 (46.5)	3 (3.2)	8 (4.5)
Do you start children on TB treatment without laboratory confirmation?					
Never	21 (70.0)	81 (81.8)	41 (41.4)	54 (58.1)	65 (36.9)
Sometimes	2 (6.7)	10 (10.1)	4 (4.0)	27 (29.0)	75 (42.6)
Often	0 (0.0)	6 (6.1)	2 (2.0)	3 (3.2)	10 (5.7)
Always	1 (3.3)	1 (1.0)	1 (1.0)	8 (8.6)	17 (9.7)
Missing	6 (20.0)	1 (1.0)	51 (51.5)	1 (1.1)	9 (5.1)
Do you wear personal protective equipment before contact with children with TB or presumptive TB?					
Never	1 (3.3)	31 (31.3)	7 (7.1)	13 (14.0)	15 (8.5)
Sometimes	0 (0.0)	11 (11.1)	5 (5.1)	13 (14.0)	38 (21.6)
Often	6 (20.0)	10 (10.1)	9 (9.1)	5 (5.4)	17 (9.7)
Always	18 (60.0)	47 (47.5)	30 (30.3)	59 (63.4)	102 (58.0)
Missing	5 (16.7)	0 (0.0)	48 (48.5)	3 (3.2)	4 (2.3)
Do you provide TB preventive therapy to asymptomatic child contacts of newly diagnosed patients?					
Never	12 (40.0)	35 (35.4)	11 (11.1)	30 (32.3)	23 (13.1)
Sometimes	4 (13.3)	15 (15.2)	5 (5.1)	27 (29.0)	33 (18.8)
Often	2 (6.7)	12 (12.1)	7 (7.1)	6 (6.5)	19 (10.8)
Always	9 (30.0)	37 (37.4)	14 (14.1)	28 (30.1)	93 (52.8)
Missing	3 (10.0)	0 (0.0)	62 (62.6)	2 (2.2)	8 (4.5)

BCG = bacilli Calmette-Guérin.

In univariable analysis, the global knowledge score was significantly higher in HCWs involved in direct TB care vs. indirect TB care in all countries (almost significantly higher in Cambodia) (Table 4). This differed between DH and PHC-based HCWs in only Cameroon and Côte d’Ivoire.

**Table 4:** Childhood TB knowledge and attitudes scores per key healthcare worker characteristic and by country

**A)**									

	District			Facility level			Position in facility		
		
	District A	District B	*P* value	DH	PHC	*P* value	Direct TB care	Indirect TB care	*P* value
Global Knowledge score, median									
Cambodia	10.6	9.2	0.589	8.4	10.1	0.928	11.5	9.1	0.057
Cameroon	10.1	10.7	0.964	11.2	9.8	0.006*	10.7	8.8	0.016*
Côte d’Ivoire	7.9	7.0	0.676	8.0	6.2	0.051*	8.0	6.3	0.010*
Sierra Leone	10.1	8.1	<0.001*	8.3	9.3	0.298	9.1	7.7	0.044*
Uganda	11.7	12.6	0.098	12.8	11.7	0.065	12.7	10.9	<0.001*
Global Attitudes score, median									
Cambodia	3.3	3.3	0.835	3.2	3.4	0.261	3.2	3.4	0.458
Cameroon	2.8	2.8	0.944	2.9	2.8	0.246	2.8	2.7	0.361
Côte d’Ivoire	2.9	2.8	0.099	2.9	2.8	0.221	2.9	2.9	0.453
Sierra Leone	2.9	2.8	0.173	2.8	2.8	0.966	2.8	2.8	0.048*
Uganda	3.0	3.2	<0.001*	3.2	3.1	0.059	3.2	3.1	0.137

**B)**						
	Contract type			Experience		
	
	Non-permanent	Permanent	*P* value	<10 years	>10 years	*P* value
Global Knowledge score, median						
Cambodia	9.2	10.5	0.511	10.1	10.1	0.678
Cameroon	8.6	10.9	<0.001*	10.2	10.7	0.645
Côte d’Ivoire	6.7	8.0	0.061	7.2	8.0	0.761
Sierra Leone	8.0	9.1	0.138	8.8	9.5	0.408
Uganda	9.9	12.3	<0.001*	12.2	11.2	0.124
Global Attitudes score, median						
Cambodia	3.3	3.3	0.981	3.2	3.4	0.244
Cameroon	2.8	2.7	0.308	2.8	2.9	0.608
Côte d’Ivoire	2.9	2.9	0.680	2.9	2.9	0.855
Sierra Leone	2.8	2.9	0.156	2.8	2.8	0.436
Uganda	3.0	3.1	0.179	3.1	3.2	0.469

* Statistically significant.

In multivariable analysis, HCW characteristics independently associated with a lower global knowledge score were: practicing at PHC level, being female, involved in indirect TB care, having a non-permanent position, having no previous research experience, and working in Cambodia, Cameroon, Côte d’Ivoire, and Sierra Leone as compared to Uganda (Table 5).

**Table 5:** Healthcare workers characteristics associated with the overall knowledge score

	Mean	95% CI	*P* value
Intercept	14.23	13.52 to 14.94	<0.01
Sex			<0.01
Male	0		
Female	−1.37	−1.87 to −0.87	
Type of health facility			0.02
District hospital	0		
Primary health centre	−0.59	−1.08 to −0.10	
Position in health facility			<0.01
Direct TB care	0		
Indirect TB care	−1.41	−1.96 to −0.86	
Contract type			<0.01
Permanent staff	0		
Other (non-permanent, volunteer)	−1.50	−2.03 to −0.96	
Previous research experience			<0.01
Yes			
No	−0.88	−1.44 to −0.31	
Country			<0.01
Uganda	0		
Cambodia	−2.18	−3.22 to −1.13	
Cameroon	−1.23	−1.90 to −0.57	
Côte d’Ivoire	−3.61	−4.35 to −2.87	
Sierra Leone	−2.57	−3.26 to −1.87	

CI = confidence interval.

### Attitudes

The overall median attitude score ranged between 2.8/4 in Cameroon and 3.3/4 in Cambodia (Table 2). Scores for cognitive and behavioural attitudes were above 3.0 in all countries. Almost all HCWs strongly agreed or agreed that they themselves should be screened for TB if they had any related signs or symptoms (Table 3). However, in Cameroon, Côte d’Ivoire and Sierra Leone, half of the respondents more disagreed or strongly disagreed that any child attending outpatient department (OPD) should be systematically screened for TB. Between 78.8% of HCWs in Cameroon and Côte d’Ivoire and 93.3% in Cambodia strongly agreed or agreed that TB diagnosis is more difficult in children than in adults (Table 3). Between 25.3% in Cameroon and Côte d’Ivoire and 56.0% of HCWs in Cambodia felt reluctant to collect and test stool samples for TB diagnosis in children.

Overall attitude scores among HCWs did not significantly differ by key HCW characteristics, except for two small differences: according to position in the health facility in Sierra Leone, and according to the district in Uganda (Table 4).

### Practices

Between 13.3% in Cambodia and 34.4% of HCWs in Cameroon reported diagnosing childhood TB in their health facility a few times a week (Table 3). Between 8.0% of HCWs in Uganda and 36.4% in Cameroon reported often/always referring children to higher levels of care for sample collection (Table 3). The proportion of HCWs reporting that they never treated children for TB in their facility without laboratory confirmation ranged from 36.9% in Uganda to 81.8% in Cameroon. Except in Uganda (52.8%), small proportions of HCWs reported providing TPT to asymptomatic child contacts of newly diagnosed TB patients. Differences in practices according HCW characteristics are given in Supplementary Data S4.7.

## DISCUSSION

This multi-country KAP survey showed limited knowledge regarding childhood TB, rather positive attitudes towards the disease and lack of experience in childhood TB management among HCWs working in DH and PHCs in resource-limited and high TB burden countries. Overall, this KAP survey confirmed and illustrated the well-known yet often unmeasured lack of knowledge and experience in childhood TB management at peripheral levels of care.

Limited practice of childhood TB diagnosis can be explained by the fact that a significant proportion of HCWs who participated in the KAP survey belonged to PHCs where childhood – or even adult – TB care services are often not available. Access to TB diagnosis for children is usually available at DHs, although only Cameroon reported a higher proportion of HCWs diagnosing children with TB or presumptive TB a few times a week in DHs vs. PHCs in this study. It is possible that the large proportion of HCWs who reported that they had never initiated children on TB treatment without laboratory confirmation is a direct consequence of this limited diagnosis experience. This is also reflected in the fact that HCWs would rather rely on “objective” laboratory findings than on their own “subjective” clinical experience.

Global knowledge scores in all countries except Côte d’Ivoire were slightly above average (9/18). Scores were significantly higher when HCWs were involved in direct TB care. These findings are concordant with other studies where frontline HCWs (physicians, nurses) had higher TB knowledge levels than other types of HCWs.^24^,^25^ Furthermore, knowledge scores were higher among HCWs with permanent contracts, possibly because they are often prioritised for any additional training sessions. In a context where staff turnover and task-shifting are common, TB sensitisation and training activities at facility-level should target all staff as far as possible, whether or not they are permanent, and whether or not they are officially involved in direct TB care, as this will help maximise awareness among care personnel who come in contact with children with presumptive TB at various entry points of the facility.

In our survey, knowledge scores on diagnosis were lowest, which is both worrying and understandable in the context of global underdiagnosis, the complexity of paediatric TB diagnosis and competing demands on HCWs’ time and expertise, especially at the PHC level. Poor knowledge of Xpert testing among HCWs in all countries may reflect the limited availability of Xpert at decentralised levels in resource-limited settings and lack of training in Xpert testing. Knowledge of HCWs on TB treatment duration in children was quite good: although low-level health facilities lack childhood TB diagnostic tools, HCWs at PHCs may distribute TB drugs to children initiated on TB treatment at referral sites. Overall, more than two-thirds of respondents in our study reported insufficient training in childhood TB, which likely explains some of the poor knowledge levels observed.

Global knowledge scores among HCWs largely differed by country, with significantly higher scores in Uganda. This may be related to the different levels of integration and decentralisation of childhood TB services in peripheral health facilities, in line with the different national strategies and in the face of many structural barriers.

Global attitudes among HCWs were systematically above average, indicating the willingness and preparedness of HCWs to diagnose and manage childhood TB in their healthcare facility. Most HCWs were favourable to systematically screen for TB all children attending OPD, except in Côte d’Ivoire; this may be related to the health system structure in Côte d’Ivoire, which has a vertical and independent network of TB centres that care mostly for adult patients. A significant proportion of HCWs were reluctant to collect stool from children, although this is now recommended by the WHO for TB molecular testing. This possibly reflects an aversion to handling stool, poor knowledge of stool as being an adequate sample to test for childhood TB or possibly, a lack of practice in line with country-specific guidance on sample collection at the time.^4^

This survey had several limitations. First, the convenience recruitment strategy led to very different sample size in each country; the small number of participants in Cambodia suggests that results should be interpreted with caution. Second, certain results may be biased due to social desirability in the context of a future project launch and the group KAP questionnaire-filling sessions. Third, some participants may have responded to the questions on the assessment of the cognitive dimension of attitudes as if these were factual or policy-based questions. Fourth, as practices were self-reported and not measured by observation, good practices among HCWs may have been overestimated. Finally, several practices questions were not adapted to HCWs with limited involvement in TB care; it should be noted that the assessment of individual practices in a context of highly diverse professions and settings is complex. An implementation component of the TB-Speed Decentralisation Study will further investigate childhood TB management practices and care pathways through observations and individual interviews.

Overall, this multi-country KAP survey involving HCWs from different levels of the healthcare system and care cascade provides valuable insight into the important knowledge gaps in childhood TB at the district level in high TB burden countries. These survey results indicate crucial next steps in the decentralisation of child TB diagnosis, including the continued improvement of the capacities and skills of HCW to detect, diagnose and treat TB in children; and improving access to simple, quick and effective diagnostic tools for children.
