# Supplementary material for: Knowledge, attitudes and practices on childhood TB among healthcare workers
Source: Int J Tuberc Lung Dis. 2022 Mar 1;26(3):243–51. doi: 10.5588/ijtld.21.0317 (PMC9121838; doi:10.5588/ijtld.21.0317)
Supplement: Supplementary file 1 [file iutld_ijtld_21.0317_supplementarydata_1.pdf]

0317 SUPPLEMENTARY DATA

Knowledge, Attitudes and Practices on childhood tuberculosis among Health Care Workers in high TB incidence countries

- Supplementary material 1. TB-Speed Decentralization Study group
- Supplementary material 2. KAP questionnaire
- Supplementary material 3. Composition of sub-scores
- Supplementary material 4. Detailed KAP results

Supplementary Data 1. TB-Speed Decentralization Study Group

Institut Pasteur du Cambodge, Epidemiology and Public Health Unit, Phnom Penh, Cambodia: BORAND Laurence, DE LAUZANNE Agathe, DIM Bunnet, FERHI Romyka, HEANG Seyla, KAING Sanary, KEANG Chanty, LY Socheat, MEAS Pichpiseth, NHOUENG Sovann, PRING Long, SRENG Vouchleang, YIN Song

National Center for Tuberculosis and Leprosy Control, Phnom Penh, Cambodia : MAO Tan Eang

IRD, Yaoundén Cameroon: AMBOUA SCHOUAME Audrey, BABEY Clifford, EDEN NGU Masama, KWEDI NOLNA Sylvie, MBANG MASSOM Douglas, MELINGUI Bernard Fortune, NGA ELOMO Nadia, GEUNOU Etienne

Programme National de Lutte contre la Tuberculose (PNLT), Yaoundé, Cameroon: MBASSA Vincent

Mother and child Centre, Chantal Biya Foundation, Yaoundé, Cameroon: TAGUEBUE Jean-Voisin

PACCI, Abidjan, Côte d'Ivoire: AKA BONY Roger, BANGA Marie-France, BOUZIÉ Olivier, DANHO Serge, DION Alphonse, GOGOUA Saulé Melissa, KOMENA Auguste Eric, KOUADIO Christian, KOUAME Abel Arkason, MOH Raoul, NGUESSAN Marcelle Sandrine, SILOUÉ Bertine, YAO Yapi Cyrille Prisca

Programme National de Lutte contre la Tuberculose (PNLT), Abidjan, Côte d'Ivoire: KOUAKOU Jacquemin

University of Bordeaux, Inserm, Institut de Recherche pour le Développement (IRD), UMR 1219, Bordeaux, France: BALESTRE Eric, BEUSCART Aurelie, FONT Hélène, JOSHI Basant, KOSKAS Nicolas, MARCY Olivier, OCCELLI Estelle, ORNE-GLIEMANN Joanna, RAZAFIMANANTSOA Manoa, VERNOUX Elodie

University of Montpellier, IRD, –INSERM, TRANSVIH MI, Montpellier, France: BONNET Maryline, CHAUVET Savine, LOUNNAS Manon

Solthis, Paris, France: BRETON Guillaume

TeAM SPI, France: NORVAL Pierre-Yves

Instituto Nacional de Saúde, Maputo, Mozambique: CASSY Sheyla, CUMBE Saniata, MATSINHE Mércia, KHOSA Celso, LIMA Yara, MABOTE Nairo, MACHAVA Salvador, MACHONISSE Emelva, ZITA Alcina, CHIÚLE Válter

Solthis, Freetown, Sierre Leone: BEYAN Prince, FLOMO Benjamin M., JALLOH Joseph Abubakarr, KAMARA Ishmael, KOROMA Monica G, LAMIN Mohamed, MATATA Lena, MUGISHA Jacob Ross, SESAY Ishmael, SENESIE Christiana M.

Ola During Children's Hospital, Freetown, Sierra Leone: MUSTAPHA Ayeshatu

National Leprosy and TB Control Programme (NLTCP), Freetown, Sierra Leone: FORAY Lynda

MUJHU Research Collaboration, MU-JHU Care Limited, Kampala, Uganda: AGONDEZE Sandra, KOBUSINGYE Agnes, NAMFUKA Mastula, WOBUDEYA Eric

Epicentre Mbarara Research Centre, Mbarara, Uganda: ARINAITWE Rinah, ABOK Florence, KAITANO Rodney, KAKURU Ronald, KASUJJA Martin, MWANGA-AMUMPAIRE Juliet, MWESIGWA Evans, NAKAZZI Claire, NATUKUNDA Naome, NUWAMANYA Simpson, NYANGOMA Miria, OGWANG Charles, ORIKIRIZA Patrick, TUMWIJUKYE Johnbosco, TURYASHEMERERWA Esther

National Tuberculosis and Leprosy Program, Kampala, Uganda: SEKADDE Moorine, TURYAHABWE Stavia

Scientific Advisory Board: CHABALA Chishala (University of Zambia), CUEVAS Luis (Liverpool School of Tropical Medicine, UK), DELACOURT Christophe (Hôpital Necker-Enfants Malades, France), GRAHAM Steve (Chair; University of Melbourne, Melbourne, Australia), GRZEMSKA Malgorzata (WHO, Switzerland), HESSELING Anneke (Stellenbosch University, Cape Town, South Africa), MALECHE-OBIMBO Elizabeth (University of Nairobi, Kenya), MUSOKE Philippa (Makerere University, Uganda), NICOL Mark (University of Western Australia, Perth, Australia).

Supplementary Data 2. KAP questionnaire on childhood TB*

**Also available in French, Khmer and Portuguese*

We developed the KAP questionnaire following a 4-step process: 1) literature review of KAP surveys on TB/childhood TB, and available questionnaires; 2) not able to identify prior KAP surveys specifically on childhood TB, design a draft questionnaire within a multidisciplinary writing group (medical epidemiologist, social scientist, statistician/psychologist and public health intern); 3) review from an external panel including three international TB experts and three country investigators; and 4) pre-testing of the questionnaire among 10 HCWs in a Uganda non-study PHC in June 2019.

Health Care Worker Information

1. What is your gender? ☐ Male ☐ Female
2. What is your age? Please write your birth date and/or your age in years.
   |___|___|___|___| / |___|___| AND/OR Age: |___|___| years
   Year *Month*
3. In what type of health facility do you currently work?
   - ◻ District Hospital
   - ◻ Primary Health Centre
4. What is your position within this health facility??
   - ◻ Community Health Worker/volunteer
   - ◻ Nursing assistant
   - ◻ Nurse
   - ◻ Midwife
   - ◻ Clinical Officer or Clinical Health Officer or Medical Assistant
   - ◻ General Practitioner
   - ◻ Paediatrician
   - ◻ Radiologist
   - ◻ Radiology Technician
   - ◻ Laboratory Technician or microscopist
   - ◻ Social assistant or counsellor
   - ◻ Other (specify) ……………………………………………………………….
5. What is your contractual situation?
   - ◻ Permanent staff
   - ◻ Temporary staff on short-term contract or locum
   - ◻ Volunteer
   - ◻ Other (specify) …………………………………………………….
6. How long have you worked within this health facility?
   - ◻ Less than 1year
   - ◻ 1 to 5 years
   - ◻ 5 to 10 years
   - ◻ More than 10 years
7. In the last 2 years, did you receive any training on childhood TB? ☐ No ☐ Yes
8. Have you ever been sick with TB yourself? ☐ No ☐ Yes

9. Has someone you are in close contact with ever been treated for TB (for example: family members, spouse, etc.)? ☐ No ☐ Yes
10. Have you already worked or participated in a research study before? ☐ No ☐ Yes

Knowledge

11. What causes TB?
    - ☐ A bacteria
    - ☐ A virus
    - ☐ A fungus
12. How does TB spread?
    - ☐ Sexually
    - ☐ Through the air, when a person with TB coughs, speaks, or sings
    - ☐ By direct contact (shaking hand, kissing, sharing food, touching surfaces with *M. tuberculosis*)
    - ☐ By sharing needles
13. Among people who are in contact with someone with TB disease *(tick all that apply):*
    - ☐ All will develop TB disease quickly
    - ☐ Some can be infected by TB without developing the disease
    - ☐ All are protected from TB
    - ☐ Some will develop TB disease quickly
    - ☐ Some may develop TB disease years later
14. Which groups of children are at high risk of developing TB? (*tick all that apply*)
    - ☐ Children aged less than 1 year
    - ☐ Children living with HIV
    - ☐ Children aged 5-10 years
    - ☐ Children with severe acute malnutrition
15. What are common TB symptoms in children? (*tick all that apply*)
    - ☐ Cough for more than 2 weeks
    - ☐ Diarrhea
    - ☐ Persistent fever
    - ☐ Loss of appetite
    - ☐ Headache
    - ☐ Night sweats
    - ☐ Cutaneous rash
    - ☐ Weight loss
16. How is TB confirmed in adults? (*tick all that apply*)
    - ☐ Blood culture
    - ☐ Skin test
    - ☐ Blood rapid test
    - ☐ Chest radiography (CXR)
    - ☐ Sputum microscopy or Xpert MTB/RIF
    - ☐ Culture
17. How is TB diagnosed in children? *(tick all that apply)*
    - ☐ Blood culture
    - ☐ Chest X-ray
    - ☐ Skin test
    - ☐ Blood rapid test

- ◻ Clinical diagnosis
- ◻ Xpert MTB/RIF or Xpert Ultra on bacteriological sample
- ◻ Culture

18. What kind of bacteriological samples are possible for TB diagnosis in children? *(tick all that apply)*
    - ◻ Expectorated sputum
    - ◻ Urine
    - ◻ Gastric aspirate
    - ◻ Nasopharyngeal aspirate
    - ◻ Cerebrospinal fluid
    - ◻ Stool samples
    - ◻ Induced sputum
    - ◻ Blood samples
19. What is the Xpert MTB/RIF test?
    - ◻ A test for CD4 lymphocytes count
    - ◻ A type of X-ray to diagnose TB
    - ◻ A molecular test for TB
    - ◻ A skin tuberculosis test
20. How many drugs are used in the initial (intensive) phase of TB treatment in children?
    - ◻ 1 or 2
    - ◻ 3 or 4
    - ◻ 5 or 6
    - ◻ 7 or 8
21. What drug(s) are parts of the initial (intensive) phase of tuberculosis treatment in children? *(tick all that apply)*
    - ◻ Isoniazid (INH)
    - ◻ Rifampicin (RIF)
    - ◻ Ciprofloxacin (CIP)
    - ◻ Levofloxacin (LEV)
    - ◻ Capreomycin (CAP)
    - ◻ Kanamycin (KAN)
    - ◻ Ethambutol (EMB)
    - ◻ Pyrazinamide (PZA)
    - ◻ Amikacin (AMK)
22. How long is the total duration of TB treatment in children?
    - ◻ 2 weeks
    - ◻ 2 months
    - ◻ 4 months
    - ◻ 6 months
    - ◻ 12 months
23. What could be the possible consequences of not completing TB treatment in children?
    - ◻ Development of resistant tuberculosis
    - ◻ Failure to fully cure the disease
    - ◻ Further transmission of the disease
    - ◻ All of the above
24. In children, TB is frequently associated with (*tick all that apply):*
    - ◻ Asthma
    - ◻ HIV
    - ◻ Malaria

- □ Severe Acute Malnutrition
- □ Diabetes

25. What is the name of the vaccine for TB?
    - □ ___________________________
    - □ I don't know
26. Does the vaccine protect fully against all forms of TB?
    - □ Yes
    - □ No
27. What is contact tracing? *(tick all that apply)*
    - □ Searching for people who were living in the same household with a patient diagnosed with TB (index case)
    - □ Searching for people who were in regular contact (school, work…) with a patient diagnosed with TB (index case)
    - □ Searching for people who were living in the neighbourhood a patient diagnosed with TB (index case)
    - □ Searching for anybody who has met at least once a patient diagnosed with TB (index case)
28. Who should receive TB preventive treatment among those following groups? *(tick all that apply)*
    - □ Children under 5 years old with a history of contact with TB
    - □ Children over 5 years old, adolescent and adults with a history of contact with TB
    - □ HIV-negative adults with a history of contact with TB
    - □ Children living with HIV without TB symptoms
    - □ Adults with HIV with TB symptoms

C. Attitudes

29. I am worried about being in contact with patients with TB
    - □ Strongly agree
    - □ Agree
    - □ Disagree
    - □ Strongly disagree
30. If I have TB symptoms or signs, I should be screened for TB
    - □ Strongly agree
    - □ Agree
    - □ Disagree
    - □ Strongly Disagree
31. If I have TB symptoms or signs, I should wear a mask at the health facility
    - □ Strongly agree
    - □ Agree
    - □ Disagree
    - □ Strongly disagree
32. If I have TB symptoms or signs, I should wear a mask/scarf at home
    - □ Strongly agree
    - □ Agree
    - □ Disagree
    - □ Strongly disagree
33. Any child attending outpatient clinics should be systematically screened for TB
    - □ Strongly agree

- ☐ Agree
- ☐ Disagree
- ☐ Strongly disagree

34. I would not accept to examine a child with suspected TB
    - ☐ Strongly agree
    - ☐ Agree
    - ☐ Disagree
    - ☐ Strongly disagree
35. I believe TB diagnosis in children is more difficult than in adults
    - ☐ Strongly agree
    - ☐ Agree
    - ☐ Disagree
    - ☐ Strongly Disagree
36. I feel reluctant to collect induced sputum / throat aspirates (Nasopharyngeal Aspirates) among children
    - ☐ Strongly agree
    - ☐ Agree
    - ☐ Disagree
    - ☐ Strongly Disagree
37. I feel reluctant to collect and test stools for TB in children
    - ☐ Strongly agree
    - ☐ Agree
    - ☐ Disagree
    - ☐ Strongly Disagree
38. Invasive methods can be used in children to diagnose TB
    - ☐ Strongly agree
    - ☐ Agree
    - ☐ Disagree
    - ☐ Strongly Disagree
39. Gastric aspirate is an invasive method to use in children
    - ☐ Strongly agree
    - ☐ Agree
    - ☐ Disagree
    - ☐ Strongly Disagree
40. I would recommend to stop treatment if a child with TB is feeling better
    - ☐ Strongly agree
    - ☐ Agree
    - ☐ Disagree
    - ☐ Strongly disagree
41. Traditional medicine can be used in children treated for TB
    - ☐ Strongly agree
    - ☐ Agree
    - ☐ Disagree
    - ☐ Strongly Disagree
42. It is important to conduct contact investigation to identify children with TB or at risk of TB
    - ☐ Strongly agree
    - ☐ Agree
    - ☐ Disagree

- ◻ Strongly disagree

43. Providing TB preventive therapy to children is important
    - ◻ Strongly agree
    - ◻ Agree
    - ◻ Disagree
    - ◻ Strongly disagree

Satisfaction with quality of care provided in the health facility

44. The majority of staff in my health facility have adequate training regarding childhood TB
    - ◻ Strongly agree
    - ◻ Agree
    - ◻ Disagree
    - ◻ Strongly Disagree
45. Diagnostic tools available in my health facility are adequate for the diagnosis of childhood TB
    - ◻ Strongly agree
    - ◻ Agree
    - ◻ Disagree
    - ◻ Strongly Disagree
46. Laboratory services in my health facility or that my health facility uses are adequate for the diagnosis of childhood TB
    - ◻ Strongly agree
    - ◻ Agree
    - ◻ Disagree
    - ◻ Strongly Disagree
47. In my health facility, laboratory test results are available on time to be able to decide to treat children for tuberculosis
    - ◻ Strongly agree
    - ◻ Agree
    - ◻ Disagree
    - ◻ Strongly Disagree
48. In my health facility, child-friendly fixed dose combination formulations are always available
    - ◻ Strongly agree
    - ◻ Agree
    - ◻ Disagree
    - ◻ Strongly disagree
49. In this health centre, it is complicated to refer children with presumptive tuberculosis to another health centre
    - ◻ Strongly agree
    - ◻ Agree
    - ◻ Disagree
    - ◻ Strongly Disagree

Community perceptions

50. People in my community believe that a child who has persistent cough should be brought to the clinic as soon as possible
    - ◻ Strongly agree
    - ◻ Agree
    - ◻ Disagree

- ◻ Strongly Disagree

51. People in my community are worried about being in contact with children with TB or presumptive TB
    - ◻ Strongly agree
    - ◻ Agree
    - ◻ Disagree
    - ◻ Strongly Disagree
52. People in my community are aware of the availability of tuberculosis services in this health centre
    - ◻ Strongly agree
    - ◻ Agree
    - ◻ Disagree
    - ◻ Strongly Disagree

D. Practices

53. How often do you diagnose children with TB or presumptive TB in your health facility?
    - ◻ Few times a week
    - ◻ Few times a month
    - ◻ Few times a year
    - ◻ Never
54. To collect sputum for TB diagnosis in a child:
    a) You give a container to the mother and you tell her to bring it back with sputum
    - ◻ Always
    - ◻ Often
    - ◻ Sometimes
    - ◻ Never

    b) You try to collect sputum on the spot/immediately
    - ◻ Always
    - ◻ Often
    - ◻ Sometimes
    - ◻ Never

    c) You refer to another center for sputum collection
    - ◻ Always
    - ◻ Often
    - ◻ Sometimes
    - ◻ Never

    d) You don’t collect sputum from children
    - ◻ Always
    - ◻ Often
    - ◻ Sometimes
    - ◻ Never
55. When a child has presumptive TB and is not able to expectorate sputum:
    a) You perform gastric aspirate
    - ◻ Always
    - ◻ Often
    - ◻ Sometimes
    - ◻ Never

    b) You perform nasopharyngeal aspirate

- ◻ Always
- ◻ Often
- ◻ Sometimes
- ◻ Never

c) You induce expectoration by nebulized hypertonic saline solution
- ◻ Always
- ◻ Often
- ◻ Sometimes
- ◻ Never

d) You collect stool sample
- ◻ Always
- ◻ Often
- ◻ Sometimes
- ◻ Never

e) You don't collect sputum
- ◻ Always
- ◻ Often
- ◻ Sometimes
- ◻ Never

f) You refer the child to higher level health facility
- ◻ Always
- ◻ Often
- ◻ Sometimes
- ◻ Never

56. Do you ask for chest X-Rays in children with presumptive TB ?
- ◻ Never
- ◻ Only if respiratory samples are negative
- ◻ Case by case, according to children's condition
- ◻ Systematically in all children

57. Do you use the following methods to perform a specimen collection procedure in a child?

a) Restrain the child during the procedure
- ◻ Always
- ◻ Often
- ◻ Sometimes
- ◻ Never

b) Take time to comfort and explain the procedure to the child
- ◻ Always
- ◻ Often
- ◻ Sometimes
- ◻ Never

c) Involve parents during the procedure
- ◻ Always
- ◻ Often
- ◻ Sometimes
- ◻ Never

58. Do you wear personal protective equipment before contact with children with TB or presumptive TB?
- ◻ Always
- ◻ Often

- ◻ Sometimes
- ◻ Never

59. How frequently do you initiate children on TB treatment?
    - ◻ A few times a week
    - ◻ A few times a month
    - ◻ A few times a year
    - ◻ Never
60. Do you prescribe a course of antibiotics in children with presumptive TB?
    - ◻ Never
    - ◻ Only if respiratory samples are negative
    - ◻ Case by case, according to children's condition
    - ◻ Systematically in all children
61. Do you start children on TB treatment without laboratory confirmation?
    - ◻ Always
    - ◻ Often
    - ◻ Sometimes
    - ◻ Never
62. What do you do if a child has yellowness of the eyes/skin after three weeks of TB treatment?
    a) You stop treatment
    - ◻ Always
    - ◻ Often
    - ◻ Sometimes
    - ◻ Never

    b) You evaluate level of liver enzymes if possible
    - ◻ Always
    - ◻ Often
    - ◻ Sometimes
    - ◻ Never

    c) You refer the child to facility with paediatrician/specialist
    - ◻ Always
    - ◻ Often
    - ◻ Sometimes
    - ◻ Never
63. Do you give advice/education to parents/guardians on what to do when the child throws up the medicine?
    - ◻ Always
    - ◻ Often
    - ◻ Sometimes
    - ◻ Never
64. When an adult patient has TB, do you recommend to avoid contact in the household with young children?
    - ◻ Always
    - ◻ Often
    - ◻ Sometimes
    - ◻ Never
65. Do you recommend to children with TB or presumptive TB to wear a mask when waiting at the health facility?
    - ◻ Always

- ◻ Often
- ◻ Sometimes
- ◻ Never

66. Do you organize or participate in TB awareness or education sessions in your health facility?
    - ◻ Always
    - ◻ Often
    - ◻ Sometimes
    - ◻ Never
67. Do you do investigation of contacts of a child newly diagnosed with TB?
    - ◻ Always
    - ◻ Often
    - ◻ Sometimes
    - ◻ Never
68. Do you do child contact tracing for newly diagnosed adult TB cases (index case)?
    - ◻ Always
    - ◻ Often
    - ◻ Sometimes
    - ◻ Never
69. Do you provide TB Preventive Therapy to asymptomatic child contacts of newly diagnosed patients?
    - ◻ Always
    - ◻ Often
    - ◻ Sometimes
    - ◻ Never

A child aged 3 years comes in with a persistent cough of 16 days duration, the cough is dry and the child is not short of breath. According to his mother, the child is less playful, looks very tired and has been feeling the same way for the past four weeks.

70. What likely diagnosis do you suspect?
    - ◻ HIV
    - ◻ Acute respiratory infection/pneumonia
    - ◻ Asthma
    - ◻ Tuberculosis
71. How would you confirm your likely diagnosis?
    - ◻ Ask for recent contact with TB patient
    - ◻ Clinical evaluation
    - ◻ HIV test
    - ◻ Xpert test
    - ◻ Malaria RTD
    - ◻ Tuberculin Skin Test
    - ◻ X-ray

A 6-year old child presents at the health facility after 1 week of antibiotics. He's feeling weak and he has been coughing for 4 weeks now. The mother explains that she came 10 days ago and that a test was done, it was negative. In your register you see that a sputum smear was done and it was negative.

72. Do you refer the child for chest-X-Ray?
    - ◻ Always
    - ◻ Often
    - ◻ Sometimes

- ◻ Never

73. Do you send the child home with a new course of antibiotics?
    - ◻ Always
    - ◻ Often
    - ◻ Sometimes
    - ◻ Never
74. Do you try to do another TB test/refer for another TB test?
    - ◻ Always
    - ◻ Often
    - ◻ Sometimes
    - ◻ Never

Supplementary Data 3. Composition of knowledge and attitudes sub-scores

	Number of questions	Question numbers
**Knowledge sub-scores**		
Epidemiology	5	11-14, 24
Diagnosis	5	15-19
Treatment	4	20-23
Prevention	4	25-28
**Attitudes sub-scores**		
Cognitive	7	30,33,35,38,41-43
Emotional	4	29,34,36,37
Behavioural	4	31,32,39,40

Supplementary Data 4. Detailed KAP results

Supplementary Table S4.1. HCW characteristics

	Cambodia (N= 30) n (%)	Cameroon (N= 99) n (%)	Côte d'Ivoire (N= 99) n (%)	Sierra Leone (N= 93) n (%)	Uganda (N= 176) n (%)
**Gender**					
Male	16 (53.3)	37 (37.4)	59 (59.6)	45 (48.4)	73 (41.5)
Female	14 (46.7)	62 (62.6)	39 (39.4)	48 (51.6)	96 (54.5)
Missing	0 (0.0)	0 (0.0)	1 (1.0)	0 (0.0)	7 (4.0)
**Age (Years) – Median**	40.0 [25.0, 62.0]	31.0 [19.9, 56.0]	36.1 [18.0, 73.6]	36.0 [24.0, 63.5]	35.3 [22,63]
Missing	0 (0.0)	0 (0.0)	3 (3.0)	20 (21.5)	1 (0.6)
**Type of Health Facility**					
District Hospital	11 (36.7)	30 (30.3)	54 (54.5)	53 (57.0)	74 (42.0)
Primary Health Clinic	19 (63.3)	69 (69.7)	45 (45.5)	40 (43.0)	102 (58.0)
**Position**					
Pediatrician	0 (0.0)	0 (0.0)	1 (1.0)	0 (0.0)	0 (0.0)
Clinical Officer or Medical Assistant	0 (0.0)	0 (0.0)	0 (0.0)	21 (22.6)	22 (12.5)
General Practitioner	3 (10.0)	15 (15.2)	4 (4.0)	1 (1.1)	8 (4.5)
Nurse	6 (20.0)	24 (24.2)	20 (20.2)	32 (34.4)	60 (34.1)
Nursing assistant	3 (10.0)	29 (29.3)	30 (30.3)	7 (7.5)	3 (1.7)
Midwife	11 (36.7)	4 (4.0)	0 (0.0)	0 (0.0)	21 (11.9)
Laboratory Technician or microscopist	1 (3.3)	13 (13.1)	12 (12.1)	20 (21.5)	25 (14.2)
Radiologist	0 (0.0)	0 (0.0)	0 (0.0)	0 (0.0)	0 (0.0)
Radiology Technician	2 (6.7)	0 (0.0)	5 (5.1)	4 (4.3)	0 (0.0)
Social assistant or counsellor	0 (0)	1 (1.0)	1 (1.0)	1 (1.1)	10 (5.7)
Community Health Worker/Volunteer	2 (6.7)	7 (7.1)	13 (13.1)	4 (4.3)	11 (6.2)
Other	2 (6.7)	6 (6.1)	12 (12.1)	3 (3.2)	16 (9.1)
Missing	0 (0.0)	0 (0.0)	1 (1.0)	0 (0.0)	0 (0.0)
**Position recoded**					
Direct TB Care	12 (40.0)	68 (68.7)	56 (56.6)	63 (67.7)	96 (54.5)
Indirect TB Care	18 (60.0)	31 (31.3)	43 (43.4)	30 (32.3)	80 (45.5)
**Contract type**					
Permanent	22 (73.3)	50 (50.5)	41 (41.4)	61 (65.6)	144 (81.8)
Temporary	4 (13.3)	23 (23.2)	8 (8.1)	2 (2.2)	17 (9.7)
Volunteer	0 (0)	20 (20.2)	19 (19.2)	30 (32.3)	12 (6.8)
Other	4 (13.3)	5 (5.1)	15 (15.2)	0 (0.0)	1 (0.6)
Missing	0 (0.0)	1 (1.0)	16 (16.2)	0 (0.0)	1 (0.6)
**Experience**					
Less than 1 year	0 (0.0)	22 (22.2)	21 (21.2)	13 (14.0)	27 (15.3)

1 to 5 years	6 (20.0)	50 (50.5)	34 (34.3)	43 (46.2)	94 (53.4)
5 to 10 years	8 (26.7)	11 (11.1)	21 (21.2)	26 (28.0)	38 (21.6)
More than 10 years	16 (53.3)	16 (16.2)	18 (18.2)	11 (11.8)	14 (8.0)
Missing	0 (0.0)	0 (0.0)	5 (5.1)	0 (0.0)	3 (1.7)
**Training in childhood TB (last 2 years)**					
No	24 (80.0)	80 (80.8)	82 (82.8)	74 (79.6)	136 (77.3)
Yes	6 (20.0)	19 (19.2)	11 (11.1)	18 (19.4)	39 (22.2)
Missing	0 (0.0)	0 (0.0)	6 (6.1)	1 (1.1)	1 (0.6)
**Ever been sick with TB**					
No	30 (100)	97 (98.0)	80 (80.8)	90 (96.8)	170(96.6)
Yes	0 (0.0)	1 (1.0)	15 (15.2)	1 (1.1)	4 (2.3)
Missing	0 (0.0)	1 (1.0)	4 (4.0)	2 (2.2)	2 (1.1)
**Close contacts treated with TB**					
No	25 (83.3)	74 (74.7)	80 (80.8)	56 (60.2)	149 (84.7)
Yes	5 (16.7)	25 (25.3)	15 (15.2)	25 (26.9)	26 (14.8)
Missing	0 (0.0)	0 (0.0)	4 (4.0)	12 (12.9)	1 (0.6)
**Research experience**					
No	15 (50.0)	64 (64.6)	72 (72.7)	63 (67.7)	107 (60.8)
Yes	11 (36.7)	35 (35.4)	7 (7.1)	17 (18.3)	42 (23.9)
Missing	4 (13.3)	0 (0.0)	20 (20.2)	13 (14.0)	27 (15.3)

Supplementary Table S4.2. Knowledge of childhood TB among Health Care Workers

	Cambodia n (%)	Cameroon n (%)	Cote d'Ivoire n (%)	Sierra Leone n (%)	Uganda n (%)
**Knowledge**					
**What causes TB?**					
A bacteria	23 (76.7)	85 (85.9)	81 (81.8)	79 (84.9)	154 (87.5)
A virus	5 (16.7)	13 (13.1)	10 (10.1)	11 (11.8)	18 (10.2)
A fungus	0 (0.0)	0 (0.0)	0 (0.0)	1 (1.1)	0 (0.0)
Missing	2 (6.7)	1 (1.0)	8 (8.1)	2 (2.2)	4 (2.3)
Wrong	7 (23.3)	14 (14.1)	18 (18.2)	14 (15.1)	22 (12.5)
**Correct**	**23 (76.7)**	**85 (85.9)**	**81 (81.8)**	**79 (84.9)**	**154 (87.5)**
**How does TB spread?**					
Sexually	0 (0.0)	0 (0)	1 (1.0)	2 (2.2)	0 (0.0)
Through the air, when a person with TB coughs, speaks, or sings	30 (100)	84 (84.8)	89 (89.9)	89 (95.7)	174 (98.9)
By direct contact (shaking, kissing, sharing food, touching surfaces with M. tuberculosis)	0 (0.0)	14 (14.1)	5 (5.1)	1 (1.1)	2 (1.1)
By sharing needles	0 (0.0)	1 (1.0)	1 (1.0)	1 (1.1)	0 (0)
Missing	0 (0.0)	0 (0.0)	3 (3.0)	0 (0.0)	0 (0.0)
Wrong	0 (0.0)	15 (15.2)	10 (10.1)	4 (4.3)	2 (1.1)
**Correct**	**30 (100)**	**84 (84.8)**	**89 (89.9)**	**89 (95.7)**	**174 (98.9)**
**Among people who are in contact with someone with TB disease:**					
Some may develop TB disease years later	21 (70.0)	72 (72.7)	60 (60.6)	45 (48.4)	135 (76.7)
Some will develop TB disease quickly	17 (56.7)	72 (72.7)	56 (56.6)	53 (57.0)	134 (76.1)
All are protected from TB	3 (10.0)	3 (3.0)	3 (3.0)	5 (5.4)	9 (5.1)
Some can be infected by TB without developing the disease	18 (60.0)	75 (75.8)	65 (65.7)	63 (67.7)	127 (72.2)
All will develop TB disease quickly	12 (40.0)	20 (20.2)	13 (13.1)	32 (34.4)	29 (16.5)
Missing	0 (0.0)	0 (0.0)	5 (5.1)	2 (2.2)	6 (3.4)
Wrong	6 (20.0)	8 (8.1)	12 (12.1)	18 (19.4)	17 (9.7)
**Correct**	**6 (20.0)**	**39 (39.4)**	**24 (24.2)**	**21 (22.6)**	**74 (42.0)**
**Partially correct**	**18 (60.0)**	**52 (52.5)**	**63 (63.6)**	**54 (58.1)**	**85 (48.3)**
**Which groups of children are at high risk of developing TB?**					
Children with severe acute malnutrition	22 (73.3)	71 (71.7)	38 (38.4)	66 (71.0)	148 (84.1)
Children aged 5-10 years	9 (30.0)	35 (35.4)	22 (22.2)	27 (29.0)	55 (31.2)
Children with HIV	28 (93.3)	90 (90.9)	77 (77.8)	68 (73.1)	171 (97.2)
Children aged less than 10 years	8 (26.7)	48 (48.5)	29 (29.3)	31 (33.3)	114 (64.8)
Missing	0 (0.0)	0 (0.0)	8 (8.1)	0 (0.0)	2 (1.1)
Wrong	1 (3.3)	10 (10.1)	19 (19.2)	5 (5.4)	6 (3.4)

**Correct**	**3 (10.0)**	**28 (28.3)**	**7 (7.1)**	**8 (8.6)**	**66 (37.5)**
**Partially correct**	**26 (86.7)**	**61 (61.6)**	**73 (73.7)**	**80 (86.0)**	**104 (59.1)**
**In children, TB is frequently associated with:**					
Diabetes	8 (26.7)	5 (5.1)	4 (4.0)	3 (3.2)	16 (9.1)
Severe acute malnutrition	25 (83.3)	81 (81.8)	42 (42.4)	67 (72.0)	142 (80.7)
Malaria	1 (3.3)	16 (16.2)	18 (18.2)	10 (10.8)	8 (4.5)
HIV	22 (73.3)	81 (81.8)	68 (68.7)	67 (72.0)	154 (87.5)
Asthma	20 (66.7)	27 (27.3)	26 (26.3)	27 (29.0)	44 (25.0)
Missing	0 (0.0)	0 (0.0)	12 (12.1)	1 (1.1)	3 (1.7)
Wrong	13 (43.3)	20 (20.2)	44 (44.4)	20 (21.5)	23 (13.1)
**Correct**	**5 (16.7)**	**43 (43.4)**	**22 (22.2)**	**28 (30.1)**	**82 (46.6)**
**Partially correct**	**12 (40.0)**	**36 (36.4)**	**33 (33.3)**	**45 (48.4)**	**71 (40.3)**
**What are common TB symptoms in children?**					
Weight loss	28 (93.3)	96 (97.0)	74 (74.7)	44 (47.3)	169 (96.0)
Cutaneous rash	2 (6.7)	16 (16.2)	3 (3.0)	8 (8.6)	16 (9.1)
Night sweats	26 (86.7)	83 (83.8)	58 (58.6)	56 (60.2)	136 (77.3)
Headache	2 (6.7)	15 (15.2)	14 (14.1)	13 (14.0)	19 (10.8)
Loss of appetite	18 (60.0)	65 (65.7)	41 (41.4)	51 (54.8)	136 (77.3)
Persistent fever	24 (80.0)	80 (80.8)	46 (46.5)	53 (57.0)	142 (80.7)
Diarrhea	0(0.0)	5 (5.1)	2 (2.0)	10 (10.8)	15 (8.5)
Cough more than 2 weeks	29 (96.7)	92 (92.9)	88 (88.9)	86 (92.5)	168 (95.5)
Missing	0 (0.0)	0 (0.0)	4 (4.0)	0 (0.0)	0 (0.0)
Wrong	0(0.0)	1 (1.0)	7 (7.1)	5 (5.4)	0 (0.0)
**Correct**	**11 (36.7)**	**33 (33.3)**	**18 (18.2)**	**14 (15.1)**	**63 (35.8)**
**Partially correct**	**19 (63.3)**	**65 (65.7)**	**74 (74.7)**	**74 (79.6)**	**113 (64.2)**
**How is TB confirmed in adults?**					
Culture	10 (33.3)	34 (34.3)	15 (15.2)	27 (29.0)	73 (41.5)
Sputum microscopy or Xpert MTB/RIF	30 (100)	79 (79.8)	61 (61.6)	83 (89.2)	167 (94.9)
Chest X-Ray	26 (86.7)	88 (88.9)	76 (76.8)	53 (57.0)	144 (81.8)
Blood rapid test	0(0.0)	9 (9.1)	12 (12.1)	7 (7.5)	9 (5.1)
Skin test	3 (10.0)	20 (20.2)	13 (13.1)	7 (7.5)	42 (23.9)
Blood culture	3 (10.0)	14 (14.1)	6 (6.1)	12 (12.9)	12 (6.8)
Missing	0 (0.0)	0 (0.0)	8 (8.1)	1 (1.1)	2 (1.1)
Wrong	19 (63.3)	74 (74.7)	85 (85.9)	47 (50.5)	113 (64.2)
**Correct**	**1 (3.3)**	**1 (1.0)**	**0 (0.0)**	**2 (2.2)**	**3 (1.7)**
**Partially correct**	**10 (33.3)**	**24 (24.2)**	**14 (14.1)**	**44 (47.3)**	**60 (34.1)**
**How is TB diagnosed in children?**					
Culture	9 (30.0)	21 (21.2)	10 (10.1)	24 (25.8)	62 (35.2)
Xpert MTB/RIF or Xpert Ultra on bacteriological sample	22 (73.3)	80 (80.8)	58 (58.6)	65 (69.9)	160 (90.9)
Clinical diagnosis	18 (60.0)	62 (62.6)	30 (30.3)	48 (51.6)	129 (73.3)

Blood rapid test	2 (6.7)	9 (9.1)	11 (11.1)	3 (3.2)	11 (6.2)
Skin test	8 (26.7)	17 (17.2)	11 (11.1)	7 (7.5)	42 (23.9)
Chest X-Ray	18 (60.0)	76 (76.8)	69 (69.7)	53 (57.0)	132 (75.0)
Blood culture	2 (6.7)	14 (14.1)	5 (5.1)	11 (11.8)	10 (5.7)
Missing	0 (0.0)	0 (0.0)	14 (14.1)	1 (1.1)	1 (0.6)
Wrong	4 (13.3)	7 (7.1)	21 (21.2)	5 (5.4)	5 (2.8)
**Correct**	**5 (16.7)**	**8 (8.1)**	**2 (2.0)**	**4 (4.3)**	**26 (14.8)**
**Partially correct**	**21 (70.0)**	**84 (84.8)**	**76 (76.8)**	**84 (90.3)**	**145 (82.4)**
**What kind of bacteriological Samples are possible for TB diagnosis in children?**					
Blood samples	2 (6.7)	15 (15.2)	9 (9.1)	14 (15.1)	20 (11.4)
Induced sputum	12 (40.0)	61 (61.6)	33 (33.3)	38 (40.9)	115 (65.3)
Stool samples	9 (30.0)	24 (24.2)	11 (11.1)	31 (33.3)	68 (38.6)
Cerebrospinal fluid	2 (6.7)	16 (16.2)	14 (14.1)	15 (16.1)	46 (26.1)
Nasopharyngeal aspirate	18 (60.0)	61 (61.6)	24 (24.2)	39 (41.9)	100 (56.8)
Gastric aspirate	10 (33.3)	35 (35.4)	28 (28.3)	30 (32.3)	113 (64.2)
Urine	0 (0.0)	5 (5.1)	2 (2.0)	6 (6.5)	83 (47.2)
Expectorated sputum	18 (60.0)	70 (70.7)	83 (83.8)	46 (49.5)	120 (68.2)
Missing	1 (3.3)	0 (0.0)	6 (6.1)	6 (6.5)	2 (1.1)
Wrong	1 (3.3)	3 (3.0)	10 (10.1)	17 (18.3)	15 (8.5)
**Correct**	**0(0.0)**	**5 (5.1)**	**2 (2.0)**	**2 (2.2)**	**7 (4.0)**
**Partially correct**	**29 (96.7)**	**91 (91.9)**	**87 (87.9)**	**74 (79.6)**	**154 (87.5)**
**What is Xpert MTB/RIF assay?**					
A test for CD4 lymphocytes count	3 (10.0)	11 (11.1)	3 (3.0)	16 (17.2)	14 (8.0)
A type of X-Ray to diagnose TB	6 (20.0)	36 (36.4)	33 (33.3)	29 (31.2)	27 (15.3)
A molecular test for TB	15 (50.0)	47 (47.5)	21 (21.2)	36 (38.7)	120 (68.2)
A skin tuberculosis test	1 (3.3)	2 (2.0)	8 (8.1)	5 (5.4)	3 (1.7)
Missing	5 (16.7)	3 (3.0)	34 (34.3)	7 (7.5)	12 (6.8)
Wrong	15 (50.0)	52 (52.5)	78 (78.8)	57 (61.3)	56 (31.8)
**Correct**	**15 (50.0)**	**47 (47.5)**	**21 (21.2)**	**36 (38.7)**	**120 (68.2)**
**Drugs used in the initial phase of TB treatment in children**					
1 or 2	14 (46.7)	56 (56.6)	40 (40.4)	65 (69.9)	55 (31.2)
3 or 4	5 (16.7)	39 (39.4)	12 (12.1)	24 (25.8)	108 (61.4)
5 or 6	0 (0.0)	3 (3.0)	2 (2.0)	2 (2.2)	0 (0.0)
7 or 8	0 (0.0)	0 (0.0)	0 (0)	0 (0.0)	2 (1.1)
Missing	11 (36.7)	1 (1.0)	45 (45.5)	2 (2.2)	11 (6.2)
Wrong	25 (83.3)	60 (60.6)	87 (87.9)	69 (74.2)	68 (38.6)
**Correct**	**5 (16.7)**	**39 (39.4)**	**12 (12.1)**	**24 (25.8)**	**108 (61.4)**
**Drugs part of initial phase of tuberculosis treatment in children**					
Amikacin	0 (0)	9 (9.1)	2 (2.0)	3 (3.2)	1 (0.6)
Pyrazinamide	9 (30.0)	48 (48.5)	18 (18.2)	42 (45.2)	108 (61.4)

Ethambutol	8 (26.7)	46 (46.5)	11 (11.1)	38 (40.9)	103 (58.5)
Kanamycin	0 (0.0)	9 (9.1)	2 (2.0)	3 (3.2)	3 (1.7)
Capreomycin	0 (0.0)	7 (7.1)	2 (2.0)	3 (3.2)	0(0.0)
Levofloxacin	0 (0.0)	8 (8.1)	2 (2.0)	9 (9.7)	7 (4.0)
Ciprofloxacin	0 (0.0)	13 (13.1)	7 (7.1)	5 (5.4)	6 (3.4)
Rifampicin	16 (53.3)	80 (80.8)	37 (37.4)	65 (69.9)	144 (81.8)
Isoniazid	17 (56.7)	65 (65.7)	40 (40.4)	63 (67.7)	153 (86.9)
Missing	8 (26.7)	2 (2.0)	42 (42.4)	3 (3.2)	9 (5.1)
Wrong	8 (26.7)	19 (19.2)	50 (50.5)	11 (11.8)	13 (7.4)
**Correct**	**4 (13.3)**	**20 (20.2)**	**4 (4.0)**	**24 (25.8)**	**70 (39.8)**
**Partially correct**	**18 (60.0)**	**60 (60.6)**	**45 (45.5)**	**58 (62.4)**	**93 (52.8)**
**Total duration of TB treatment in children**					
2 weeks	0 (0.0)	3 (3.0)	4 (4.0)	8 (8.6)	1 (0.6)
2 months	0 (0.0)	10 (10.1)	3 (3.0)	6 (6.5)	6 (3.4)
4 months	0 (0.0)	8 (8.1)	3 (3.0)	8 (8.6)	8 (4.5)
6 months	24 (80.0)	72 (72.7)	59 (59.6)	55 (59.1)	152 (86.4)
12 months	0 (0.0)	6 (6.1)	2 (2.0)	12 (12.9)	4 (2.3)
Missing	6 (20.0)	0(0.0)	28 (28.3)	4 (4.3)	5 (2.8)
Wrong	6 (20.0)	27 (27.3)	40 (40.4)	38 (40.9)	24 (13.6)
**Correct**	**24 (80.0)**	**72 (72.7)**	**59 (59.6)**	**55 (59.1)**	**152 (86.4)**
**Possible consequences of not completing TB treatment in children**					
Development of resistant tuberculosis	6 (20.0)	38 (38.4)	34 (34.3)	19 (20.4)	33 (18.8)
Failure to fully cure the disease	3 (10.0)	12 (12.1)	20 (20.2)	9 (9.7)	7 (4.0)
Further transmission of the disease	0 (0.0)	1 (1.0)	5 (5.1)	6 (6.5)	0 (0)
All of the above	20 (66.7)	48 (48.5)	19 (19.2)	58 (62.4)	134 (76.1)
Missing	1 (3.3)	0(0.0)	21 (21.2)	1 (1.1)	2 (1.1)
Wrong	10 (33.3)	51 (51.5)	80 (80.8)	35 (37.6)	42 (23.9)
**Correct**	**20 (66.7)**	**48 (48.5)**	**19 (19.2)**	**58 (62.4)**	**134 (76.1)**
**Name of vaccine against TB**					
BCG	26 (86.7)	84 (84.8)	52 (52.5)	54 (58.1)	146 (83.0)
Wrong	4 (13.3)	15 (15.2)	47 (47.5)	39 (41.9)	30 (17.0)
**Correct**	**26 (86.7)**	**84 (84.8)**	**52 (52.5)**	**54 (58.1)**	**146 (83.0)**
**Does the vaccine protect fully against all forms of TB?**					
No	17 (56.7)	43 (43.4)	41 (41.4)	39 (41.9)	87 (49.4)
Yes	10 (33.3)	56 (56.6)	38 (38.4)	52 (55.9)	80 (45.5)
Missing	3 (10.0)	0(0.0)	20 (20.2)	2 (2.2)	9 (5.1)
Wrong	13 (43.3)	56 (56.6)	58 (58.6)	54 (58.1)	89 (50.6)
**Correct**	**17 (56.7)**	**43 (43.4)**	**41 (41.4)**	**39 (41.9)**	**87 (49.4)**
**What is Contact tracing**					
Searching for anybody who has met at least once an index case	4 (13.3)	41 (41.4)	9 (9.1)	27 (29.0)	43 (24.4)

Searching for people who are living in the neighborhood of index case	15 (50.0)	57 (57.6)	34 (34.3)	35 (37.6)	73 (41.5)
Searching for people who were regular in contact with index case	23 (76.7)	82 (82.8)	55 (55.6)	58 (62.4)	126 (71.6)
Searching for people living in same household with index case	30 (100)	85 (85.9)	71 (71.7)	71 (76.3)	165 (93.8)
Missing	0 (0.0)	0 (0.0)	15 (15.2)	3 (3.2)	2 (1.1)
Wrong	5 (16.7)	42 (42.4)	29 (29.3)	32 (34.4)	37 (21.0)
**Correct**	**9 (30.0)**	**21 (21.2)**	**19 (19.2)**	**17 (18.3)**	**41 (23.3)**
**Partially correct**	**16 (53.3)**	**36 (36.4)**	**51 (51.5)**	**44 (47.3)**	**98 (55.7)**
**Who should receive TB preventive therapy?**					
Adults with HIV with TB symptoms	19 (63.3)	42 (42.4)	32 (32.3)	37 (39.8)	71 (40.3)
Children living with HIV without TB symptoms	10 (33.3)	54 (54.5)	24 (24.2)	40 (43.0)	133 (75.6)
HIV-negative adults with a history of contact with TB	12 (40.0)	29 (29.3)	16 (16.2)	30 (32.3)	59 (33.5)
Children over 5, adolescent and adults with a history of contact with TB	12 (40.0)	41 (41.4)	23 (23.2)	40 (43.0)	68 (38.6)
Children under 5 with a history of contact with TB	26 (86.7)	77 (77.8)	58 (58.6)	61 (65.6)	144 (81.8)
Missing	2 (6.7)	2 (2.0)	16 (16.2)	3 (3.2)	3 (1.7)
Wrong	19 (63.3)	55 (55.6)	60 (60.6)	61 (65.6)	84 (47.7)
**Correct**	**2 (6.7)**	**17 (17.2)**	**2 (2.0)**	**4 (4.3)**	**38 (21.6)**
**Partially correct**	**9 (30.0)**	**27 (27.3)**	**37 (37.4)**	**28 (30.1)**	**54 (30.7)**

Supplementary Table S4.3. Attitudes regarding childhood TB among Health Care Workers

	Cambodia n (%)	Cameroon n (%)	Côte d'Ivoire n (%)	Sierra Leone n (%)	Uganda n (%)
**I am worried about being in contact with patients with TB**					
Strongly disagree	1 (3.3)	11 (11.1)	1 (1.0)	9 (9.7)	6 (3.4)
Disagree	1 (3.3)	19 (19.2)	17 (17.2)	22 (23.7)	20 (11.4)
Agree	7 (23.3)	47 (47.5)	38 (38.4)	26 (28.0)	61 (34.7)
Strongly agree	21 (70.0)	22 (22.2)	38 (38.4)	36 (38.7)	87 (49.4)
Missing	0 (0.0)	0 (0.0)	5 (5.1)	0 (0.0)	2 (1.1)
**I would not accept to examine a child with suspected TB**					
Strongly disagree	8 (26.7)	62 (62.6)	47 (47.5)	43 (46.2)	101 (57.4)
Disagree	16 (53.3)	30 (30.3)	34 (34.3)	30 (32.3)	62 (35.2)
Agree	3 (10.0)	4 (4.0)	5 (5.1)	6 (6.5)	5 (2.8)
Strongly agree	1 (3.3)	3 (3.0)	4 (4.0)	13 (14.0)	8 (4.5)
Missing	2 (6.7)	0 (0.0)	9 (9.1)	1 (1.1)	0 (0.0)
**I feel reluctant to collect induced sputum / throat aspirates (Nasopharyngeal Aspirates) among children**					
Strongly disagree	2 (6.7)	26 (26.3)	14 (14.1)	22 (23.7)	28 (15.9)
Disagree	5 (16.7)	48 (48.5)	34 (34.3)	37 (39.8)	59 (33.5)
Agree	15 (50.0)	16 (16.2)	30 (30.3)	20 (21.5)	58 (33.0)
Strongly agree	6 (20.0)	9 (9.1)	6 (6.1)	14 (15.1)	23 (13.1)
Missing	2 (6.7)	0(0.0)	15 (15.2)	0 (0.0)	8 (4.5)
**I feel reluctant to collect and test stools for TB in children**					
Strongly disagree	2 (6.7)	23 (23.2)	10 (10.1)	24 (25.8)	37 (21.0)
Disagree	10 (33.3)	49 (49.5)	38 (38.4)	36 (38.7)	57 (32.4)
Agree	11 (36.7)	15 (15.2)	18 (18.2)	18 (19.4)	52 (29.5)
Strongly agree	6 (20.0)	10 (10.1)	7 (7.1)	14 (15.1)	22 (12.5)
Missing	1 (3.3)	2 (2.0)	26 (26.3)	1 (1.1)	8 (4.5)
**Any child attending outpatient clinics should be systematically screened for TB**					
Strongly disagree	3 (10.0)	7 (7.1)	6 (6.1)	13 (14.0)	7 (4.0)
Disagree	1 (3.3)	35 (35.4)	40 (40.4)	33 (35.5)	30 (17.0)
Agree	6 (20.0)	39 (39.4)	22 (22.2)	24 (25.8)	44 (25.0)
Strongly agree	19 (63.3)	17 (17.2)	16 (16.2)	22 (23.7)	92 (52.3)

Missing	1 (3.3)	1 (1.0)	15 (15.2)	1 (1.1)	3 (1.7)
**I believe TB diagnosis in children is more difficult than in adults**					
Strongly disagree	0 (0.0)	7 (7.1)	2 (2.0)	4 (4.3)	9 (5.1)
Disagree	1 (3.3)	14 (14.1)	6 (6.1)	6 (6.5)	13 (7.4)
Agree	16 (53.3)	46 (46.5)	45 (45.5)	19 (20.4)	72 (40.9)
Strongly agree	12 (40.0)	32 (32.3)	33 (33.3)	63 (67.7)	80 (45.5)
Missing	1 (3.3)	0 (0.0)	13 (13.1)	1 (1.1)	2 (1.1)
**Invasive methods can be used in children to diagnose TB**					
Strongly disagree	0 (0)	5 (5.1)	4 (4.0)	7 (7.5)	9 (5.1)
Disagree	1 (3.3)	18 (18.2)	17 (17.2)	19 (20.4)	25 (14.2)
Agree	14 (46.7)	63 (63.6)	26 (26.3)	36 (38.7)	78 (44.3)
Strongly agree	9 (30.0)	12 (12.1)	11 (11.1)	27 (29.0)	47 (26.7)
Missing	6 (20.0)	1 (1.0)	41 (41.4)	4 (4.3)	17 (9.7)
**Traditional medicine can be used in children treated for TB**					
Strongly disagree	20 (66.7)	56 (56.6)	34 (34.3)	54 (58.1)	130 (73.9)
Disagree	9 (30.0)	40 (40.4)	30 (30.3)	30 (32.3)	33 (18.8)
Agree	1 (3.3)	3 (3.0)	12 (12.1)	2 (2.2)	6 (3.4)
Strongly agree	0 (0.0)	0 (0.0)	4 (4.0)	7 (7.5)	6 (3.4)
Missing	0 (0.0)	0 (0.0)	19 (19.2)	0 (0.0)	1 (0.6)
**It is important to conduct contact investigation to identify children with TB or at risk of TB**					
Strongly disagree	0 (0.0)	2 (2.0)	1 (1.0)	4 (4.3)	5 (2.8)
Disagree	1 (3.3)	1 (1.0)	3 (3.0)	2 (2.2)	7 (4.0)
Agree	11 (36.7)	41 (41.4)	35 (35.4)	29 (31.2)	41 (23.3)
Strongly agree	17 (56.7)	55 (55.6)	42 (42.4)	57 (61.3)	121 (68.8)
Missing	1 (3.3)	0 (0.0)	18 (18.2)	1 (1.1)	2 (1.1)
**Gastric aspirate is an invasive method to use in children**					
Strongly disagree	0 (0.0)	8 (8.1)	3 (3.0)	2 (2.2)	8 (4.5)
Disagree	1 (3.3)	22 (22.2)	14 (14.1)	22 (23.7)	22 (12.5)
Agree	16 (53.3)	61 (61.6)	25 (25.3)	32 (34.4)	78 (44.3)
Strongly agree	7 (23.3)	7 (7.1)	11 (11.1)	31 (33.3)	56 (31.8)
Missing	6 (20.0)	1 (1.0)	46 (46.5)	6 (6.5)	12 (6.8)
**Providing TB preventive therapy to children is important**					
Strongly disagree	0 (0.0)	6 (6.1)	5 (5.1)	3 (3.2)	2 (1.1)

Disagree	1 (3.3)	21 (21.2)	9 (9.1)	5 (5.4)	2 (1.1)
Agree	8 (26.7)	37 (37.4)	35 (35.4)	19 (20.4)	40 (22.7)
Strongly agree	20 (66.7)	35 (35.4)	30 (30.3)	65 (69.9)	131 (74.4)
Missing	1 (3.3)	0 (0.0)	20 (20.2)	1 (1.1)	1 (0.6)
**If I have TB symptoms or signs, I should be screened for TB**					
Strongly disagree	0 (0.0)	0 (0.0)	1 (1.0)	2 (2.2)	1 (0.6)
Disagree	0 (0.0)	1 (1.0)	0 (0)	3 (3.2)	0 (0.0)
Agree	3 (10.0)	31 (31.3)	35 (35.4)	16 (17.2)	26 (14.8)
Strongly agree	26 (86.7)	67 (67.7)	59 (59.6)	72 (77.4)	149 (84.7)
Missing	1 (3.3)	0 (0.0)	4 (4.0)	0 (0.0)	0 (0.0)
**lf I have TB symptoms or signs, l should wear a mask at the health facility**					
Strongly disagree	2 (6.7)	5 (5.1)	3 (3.0)	17 (18.3)	6 (3.4)
Disagree	0 (0.0)	15 (15.2)	10 (10.1)	24 (25.8)	22 (12.5)
Agree	6 (20.0)	43 (43.4)	41 (41.4)	20 (21.5)	44 (25.0)
Strongly agree	22 (73.3)	36 (36.4)	42 (42.4)	31 (33.3)	104 (59.1)
Missing	0 (0.0)	0 (0)	3 (3.0)	1 (1.1)	0 (0.0)
**lf I have TB symptoms or signs, l should wear a mask/scarf at home**					
Strongly disagree	2 (6.7)	6 (6.1)	5 (5.1)	27 (29.0)	10 (5.7)
Disagree	1 (3.3)	27 (27.3)	17 (17.2)	35 (37.6)	32 (18.2)
Agree	8 (26.7)	40 (40.4)	39 (39.4)	19 (20.4)	56 (31.8)
Strongly agree	19 (63.3)	26 (26.3)	27 (27.3)	12 (12.9)	76 (43.2)
Missing	0 (0.0)	0 (0.0)	0 (0.0)	0 (0.0)	2 (1.1)
**I would recommend to stop treatment if a child with TB is feeling better**					
Strongly disagree	17 (56.7)	50 (50.5)	28 (28.3)	44 (47.3)	113 (64.2)
Disagree	8 (26.7)	41 (41.4)	35 (35.4)	31 (33.3)	45 (25.6)
Agree	0 (0.0)	6 (6.1)	9 (9.1)	9 (9.7)	7 (4.0)
Strongly agree	2 (6.7)	2 (2.0)	7 (7.1)	9 (9.7)	9 (5.1)
Missing	3 (10.0)	0 (0.0)	20 (20.2)	0 (0.0)	2 (1.1)
**The majority of staff in my health facility have adequate training regarding childhood TB**					
Strongly disagree	3 (10.0)	13 (13.1)	16 (16.2)	27 (29.0)	48 (27.3)
Disagree	14 (46.7)	51 (51.5)	30 (30.3)	33 (35.5)	83 (47.2)
Agree	5 (16.7)	30 (30.3)	21 (21.2)	19 (20.4)	25 (14.2)
Strongly agree	6 (20.0)	5 (5.1)	9 (9.1)	13 (14.0)	18 (10.2)
Missing	2 (6.7)	0 (0.0)	23 (23.2)	1 (1.1)	2 (1.1)

**Diagnostic tools available in my health facility are adequate for the diagnosis of childhood TB**					
Strongly disagree	6 (20.0)	26 (26.3)	14 (14.1)	29 (31.2)	49 (27.8)
Disagree	17 (56.7)	48 (48.5)	26 (26.3)	33 (35.5)	72 (40.9)
Agree	4 (13.3)	20 (20.2)	19 (19.2)	20 (21.5)	34 (19.3)
Strongly agree	1 (3.3)	5 (5.1)	7 (7.1)	10 (10.8)	18 (10.2)
Missing	2 (6.7)	0 (0.0)	33 (33.3)	1 (1.1)	3 (1.7)
**Laboratory services in my health facility or that my health facility uses are adequate for the diagnosis of childhood TB**					
Strongly disagree	6 (20.0)	15 (15.2)	12 (12.1)	24 (25.8)	28 (15.9)
Disagree	17 (56.7)	50 (50.5)	19 (19.2)	23 (24.7)	75 (42.6)
Agree	2 (6.7)	27 (27.3)	24 (24.2)	26 (28.0)	50 (28.4)
Strongly agree	2 (6.7)	7 (7.1)	8 (8.1)	18 (19.4)	22 (12.5)
Missing	3 (10.0)	0 (0.0)	36 (36.4)	2 (2.2)	1 (0.6)
**In my health facility, laboratory test results are available on time to be able to decide to treat children for tuberculosis**					
Strongly disagree	3 (10.0)	15 (15.2)	8 (8.1)	16 (17.2)	14 (8.0)
Disagree	14 (46.7)	41 (41.4)	11 (11.1)	22 (23.7)	59 (33.5)
Agree	5 (16.7)	32 (32.3)	34 (34.3)	31 (33.3)	70 (39.8)
Strongly agree	4 (13.3)	11 (11.1)	13 (13.1)	23 (24.7)	31 (17.6)
Missing	4 (13.3)	0 (0.0)	33 (33.3)	1 (1.1)	2 (1.1)
**In my health facility, child-friendly fixed dose combination formulations are always available**					
Strongly disagree	0 (0.0)	11 (11.1)	5 (5.1)	12 (12.9)	6 (3.4)
Disagree	6 (20.0)	48 (48.5)	11 (11.1)	32 (34.4)	35 (19.9)
Agree	9 (30.0)	33 (33.3)	25 (25.3)	32 (34.4)	82 (46.6)
Strongly agree	8 (26.7)	5 (5.1)	9 (9.1)	15 (16.1)	39 (22.2)
Missing	7 (23.3)	2 (2.0)	49 (49.5)	2 (2.2)	14 (8.0)
**In this health centre, it is complicated to refer children with presumptive tuberculosis to another health centre**					
Strongly disagree	2 (6.7)	21 (21.2)	10 (10.1)	18 (19.4)	36 (20.5)
Disagree	7 (23.3)	49 (49.5)	24 (24.2)	32 (34.4)	71 (40.3)
Agree	12 (40.0)	25 (25.3)	19 (19.2)	23 (24.7)	39 (22.2)
Strongly agree	5 (16.7)	3 (3.0)	9 (9.1)	19 (20.4)	26 (14.8)

Missing	4 (13.3)	1 (1.0)	37 (37.4)	1 (1.1)	4 (2.3)
**People in my community believe that a child who has persistent cough should be brought to the clinic as soon as possible**					
Strongly disagree	1 (3.3)	1 (1.0)	0 (0)	4 (4.3)	15 (8.5)
Disagree	8 (26.7)	14 (14.1)	5 (5.1)	12 (12.9)	45 (25.6)
Agree	12 (40.0)	51 (51.5)	41 (41.4)	39 (41.9)	84 (47.7)
Strongly agree	8 (26.7)	32 (32.3)	40 (40.4)	37 (39.8)	31 (17.6)
Missing	1 (3.3)	1 (1.0)	13 (13.1)	1 (1.1)	1 (0.6)
**People in my community are worried about being in contact with children with TB or presumptive TB**					
Strongly disagree	0 (0.0)	5 (5.1)	5 (5.1)	6 (6.5)	4 (2.3)
Disagree	7 (23.3)	19 (19.2)	23 (23.2)	25 (26.9)	59 (33.5)
Agree	11 (36.7)	55 (55.6)	45 (45.5)	34 (36.6)	74 (42.0)
Strongly agree	12 (40.0)	19 (19.2)	11 (11.1)	27 (29.0)	38 (21.6)
Missing	0 (0.0)	1 (1.0)	15 (15.2)	1 (1.1)	1 (0.6)
**People in my community are aware of the availability of tuberculosis services in this health centre**					
Strongly disagree	1 (3.3)	6 (6.1)	2 (2.0)	3 (3.2)	3 (1.7)
Disagree	2 (6.7)	25 (25.3)	8 (8.1)	10 (10.8)	20 (11.4)
Agree	16 (53.3)	45 (45.5)	44 (44.4)	30 (32.3)	101 (57.4)
Strongly agree	8 (26.7)	22 (22.2)	24 (24.2)	45 (48.4)	49 (27.8)
Missing	3 (10.0)	1 (1.0)	21 (21.2)	5 (5.4)	3 (1.7)

Supplementary Table S4.4. Practices around childhood TB among Health Care Workers

	Cambodia n (%)	Cameroon n (%)	Côte d'Ivoire n (%)	Sierra Leone n (%)	Uganda n (%)
**Practices**					
**How often do you diagnose children with TB or presumptive TB in your health facility?**					
Never	8 (26.7)	3 (3.0)	0 (0)	9 (9.7)	9 (5.1)
Few times a year	5 (16.7)	26 (26.3)	11 (11.1)	16 (17.2)	51 (29.0)
Few times a month	9 (30.0)	24 (24.2)	17 (17.2)	31 (33.3)	60 (34.1)
Few times a week	4 (13.3)	18 (18.2)	15 (15.2)	32 (34.4)	49 (27.8)
Missing	4 (13.3)	28 (28.3)	56 (56.6)	5 (5.4)	7 (4.0)
**Do you ask for chest X-Rays in children with presumptive TB?**					
Systematically in all children	1 (3.3)	20 (20.2)	12 (12.1)	8 (8.6)	14 (8.0)
Case by case, according to children condition	7 (23.3)	34 (34.3)	16 (16.2)	42 (45.2)	62 (35.2)
Only if respiratory samples are negative	9 (30.0)	27 (27.3)	18 (18.2)	24 (25.8)	55 (31.2)
Never	10 (33.3)	15 (15.2)	3 (3.0)	19 (20.4)	33 (18.8)
Missing	3 (10.0)	3 (3.0)	50 (50.5)	0 (0)	12 (6.8)
**To collect sputum for TB diagnosis in a child**					
**You give a container to the mother and you tell her to bring it back with sputum**					
Never	10 (33.3)	40 (40.4)	9 (9.1)	29 (31.2)	35 (19.9)
Sometimes	6 (20.0)	17 (17.2)	9 (9.1)	22 (23.7)	67 (38.1)
Often	3 (10.0)	13 (13.1)	19 (19.2)	12 (12.9)	13 (7.4)
Always	8 (26.7)	28 (28.3)	21 (21.2)	28 (30.1)	58 (33.0)
Missing	3 (10.0)	1 (1.0)	41 (41.4)	2 (2.2)	3 (1.7)
**You try to collect sputum on the spot/immediately**					
Never	6 (20.0)	44 (44.4)	11 (11.1)	19 (20.4)	12 (6.8)
Sometimes	5 (16.7)	18 (18.2)	6 (6.1)	19 (20.4)	55 (31.2)
Often	2 (6.7)	17 (17.2)	16 (16.2)	14 (15.1)	22 (12.5)
Always	11 (36.7)	18 (18.2)	23 (23.2)	38 (40.9)	81 (46.0)
Missing	6 (20.0)	2 (2.0)	43 (43.4)	3 (3.2)	6 (3.4)
**You refer to another center for sputum collection**					
Never	16 (53.3)	48 (48.5)	26 (26.3)	56 (60.2)	110 (62.5)
Sometimes	4 (13.3)	14 (14.1)	12 (12.1)	19 (20.4)	44 (25.0)
Often	2 (6.7)	11 (11.1)	8 (8.1)	5 (5.4)	4 (2.3)
Always	4 (13.3)	25 (25.3)	7 (7.1)	10 (10.8)	10 (5.7)
Missing	4 (13.3)	1 (1.0)	46 (46.5)	3 (3.2)	8 (4.5)

**You don't collect sputum from children**					
Never	16 (53.3)	61 (61.6)	21 (21.2)	37 (39.8)	70 (39.8)
Sometimes	8 (26.7)	20 (20.2)	6 (6.1)	28 (30.1)	71 (40.3)
Often	2 (6.7)	8 (8.1)	12 (12.1)	9 (9.7)	9 (5.1)
Always	0 (0)	7 (7.1)	5 (5.1)	14 (15.1)	15 (8.5)
Missing	4 (13.3)	3 (3.0)	55 (55.6)	5 (5.4)	11 (6.2)
**When a child has presumptive TB and is not able to expectorate sputum**					
**You perform gastric aspirate**					
Never	25 (83.3)	56 (56.6)	17 (17.2)	19 (20.4)	71 (40.3)
Sometimes	0 (0)	9 (9.1)	5 (5.1)	30 (32.3)	45 (25.6)
Often	1 (3.3)	16 (16.2)	12 (12.1)	10 (10.8)	18 (10.2)
Always	0 (0.0)	16 (16.2)	9 (9.1)	24 (25.8)	36 (20.5)
Missing	4 (13.3)	2 (2.0)	56 (56.6)	10 (10.8)	6 (3.4)
**You perform nasopharyngeal aspirate**					
Never	24 (80.0)	51 (51.5)	28 (28.3)	38 (40.9)	99 (56.2)
Sometimes	1 (3.3)	18 (18.2)	3 (3.0)	26 (28.0)	41 (23.3)
Often	0 (0.0)	13 (13.1)	4 (4.0)	7 (7.5)	9 (5.1)
Always	1 (3.3)	14 (14.1)	4 (4.0)	14 (15.1)	17 (9.7)
Missing	4 (13.3)	3 (3.0)	60 (60.6)	8 (8.6)	10 (5.7)
**You induce expectoration by nebulized hypertonic saline solution**					
Never	21 (70.0)	65 (65.7)	29 (29.3)	41 (44.1)	102 (58.0)
Sometimes	3 (10.0)	18 (18.2)	3 (3.0)	19 (20.4)	35 (19.9)
Often	0 (0.0)	12 (12.1)	2 (2.0)	12 (12.9)	16 (9.1)
Always	1 (3.3)	2 (2.0)	5 (5.1)	14 (15.1)	7 (4.0)
Missing	5 (16.7)	2 (2.0)	60 (60.6)	7 (7.5)	16 (9.1)
**You collect stool sample**					
Never	24 (80.0)	67 (67.7)	27 (27.3)	49 (52.7)	110 (62.5)
Sometimes	1 (3.3)	7 (7.1)	3 (3.0)	20 (21.5)	35 (19.9)
Often	0 (0.0)	12 (12.1)	3 (3.0)	6 (6.5)	6 (3.4)
Always	1 (3.3)	7 (7.1)	4 (4.0)	13 (14.0)	13 (7.4)
Missing	4 (13.3)	6 (6.1)	62 (62.6)	5 (5.4)	12 (6.8)
**You don't collect sputum**					
Never	12 (40.0)	58 (58.6)	15 (15.2)	35 (37.6)	60 (34.1)
Sometimes	5 (16.7)	11 (11.1)	6 (6.1)	22 (23.7)	66 (37.5)
Often	3 (10.0)	13 (13.1)	8 (8.1)	9 (9.7)	9 (5.1)
Always	1 (3.3)	13 (13.1)	3 (3.0)	21 (22.6)	27 (15.3)
Missing	9 (30.0)	4 (4.0)	67 (67.7)	6 (6.5)	14 (8.0)
**You refer the child to higher level health facility**					
Never	10 (33.3)	9 (9.1)	6 (6.1)	16 (17.2)	52 (29.5)

Sometimes	9 (30.0)	24 (24.2)	8 (8.1)	36 (38.7)	65 (36.9)
Often	3 (10.0)	17 (17.2)	14 (14.1)	8 (8.6)	9 (5.1)
Always	4 (13.3)	47 (47.5)	15 (15.2)	30 (32.3)	40 (22.7)
Missing	4 (13.3)	2 (2.0)	56 (56.6)	3 (3.2)	10 (5.7)
**Do you use the following methods to perform a specimen collection procedure in a child**					
**Restrain the child during the procedure**					
Never	18 (60.0)	36 (36.4)	11 (11.1)	28 (30.1)	85 (48.3)
Sometimes	3 (10.0)	20 (20.2)	7 (7.1)	23 (24.7)	41 (23.3)
Often	1 (3.3)	19 (19.2)	9 (9.1)	13 (14.0)	12 (6.8)
Always	1 (3.3)	22 (22.2)	11 (11.1)	20 (21.5)	22 (12.5)
Missing	7 (23.3)	2 (2.0)	61 (61.6)	9 (9.7)	16 (9.1)
**Take time to comfort and explain the procedure to the child**					
Never	11 (36.7)	23 (23.2)	5 (5.1)	21 (22.6)	33 (18.8)
Sometimes	7 (23.3)	12 (12.1)	5 (5.1)	14 (15.1)	35 (19.9)
Often	4 (13.3)	16 (16.2)	6 (6.1)	7 (7.5)	19 (10.8)
Always	2 (6.7)	47 (47.5)	21 (21.2)	46 (49.5)	80 (45.5)
Missing	6 (20.0)	1 (1.0)	62 (62.6)	5 (5.4)	9 (5.1)
**Involve parents during the procedure**					
Never	7 (23.3)	21 (21.2)	3 (3.0)	14 (15.1)	9 (5.1)
Sometimes	5 (16.7)	6 (6.1)	4 (4.0)	15 (16.1)	22 (12.5)
Often	7 (23.3)	5 (5.1)	3 (3.0)	1 (1.1)	8 (4.5)
Always	3 (10.0)	65 (65.7)	36 (36.4)	59 (63.4)	130 (73.9)
Missing	8 (26.7)	2 (2.0)	53 (53.5)	4 (4.3)	7 (4.0)
**Do you start children on TB treatment without laboratory confirmation?**					
Never	21 (70.0)	81 (81.8)	41 (41.4)	54 (58.1)	65 (36.9)
Sometimes	2 (6.7)	10 (10.1)	4 (4.0)	27 (29.0)	75 (42.6)
Often	0 (0.0)	6 (6.1)	2 (2.0)	3 (3.2)	10 (5.7)
Always	1 (3.3)	1 (1.0)	1 (1.0)	8 (8.6)	17 (9.7)
Missing	6 (20.0)	1 (1.0)	51 (51.5)	1 (1.1)	9 (5.1)
**Do you give advice/education to parents/guardians on what to do when the child throws up the medicine?**					
Never	2 (6.7)	8 (8.1)	1 (1.0)	5 (5.4)	4 (2.3)
Sometimes	1 (3.3)	6 (6.1)	2 (2.0)	11 (11.8)	11 (6.2)
Often	6 (20.0)	8 (8.1)	7 (7.1)	0 (0)	12 (6.8)
Always	17 (56.7)	76 (76.8)	38 (38.4)	76 (81.7)	147 (83.5)
Missing	4 (13.3)	1 (1.0)	51 (51.5)	1 (1.1)	2 (1.1)
**How frequently do you initiate children on TB treatment?**					

Never	5 (16.7)	34 (34.3)	5 (5.1)	10 (10.8)	15 (8.5)
A few times a year	4 (13.3)	21 (21.2)	8 (8.1)	8 (8.6)	56 (31.8)
A few times a month	6 (20.0)	29 (29.3)	12 (12.1)	35 (37.6)	48 (27.3)
A few times a week	7 (23.3)	15 (15.2)	8 (8.1)	38 (40.9)	45 (25.6)
Missing	8 (26.7)	34 (34.3)	66 (66.7)	2 (2.2)	12 (6.8)
**Do you prescribe a course of antibiotics in children with presumptive TB?**					
Systematically in all children	0 (0.0)	10 (10.1)	2 (2.0)	8 (8.6)	9 (5.1)
Case by case, according to children's condition	4 (13.3)	43 (43.4)	12 (12.1)	35 (37.6)	76 (43.2)
Only if respiratory samples are negative	9 (30.0)	16 (16.2)	13 (13.1)	19 (20.4)	53 (30.1)
Never	10 (33.3)	29 (29.3)	14 (14.1)	28 (30.1)	27 (15.3)
Missing	7 (23.3)	1 (1.0)	58 (58.6)	3 (3.2)	11 (6.2)
**What do you do if a child has yellowness of the eyes / skin after three weeks of TB treatment**					
**You stop treatment**					
Never	12 (40.0)	44 (44.4)	14 (14.1)	30 (32.3)	46 (26.1)
Sometimes	1 (3.3)	16 (16.2)	*3 (3.0)*	33 (35.5)	47 (26.7)
Often	2 (6.7)	6 (6.1)	5 (5.1)	3 (3.2)	6 (3.4)
Always	3 (10.0)	27 (27.3)	*9 (9.1)*	17 (18.3)	64 (36.4)
Missing	12 (40.0)	6 (6.1)	68 (68.7)	10 (10.8)	13 (7.4)
**You evaluate level of liver enzymes if possible**					
Never	10 (33.3)	19 (19.2)	4 (4.0)	23 (24.7)	30 (17.0)
Sometimes	0 (0)	10 (10.1)	3 (3.0)	26 (28.0)	31 (17.6)
Often	2 (6.7)	13 (13.1)	5 (5.1)	10 (10.8)	18 (10.2)
Always	4 (13.3)	54 (54.5)	18 (18.2)	28 (30.1)	84 (47.7)
Missing	14 (46.7)	3 (3.0)	69 (69.7)	6 (6.5)	13 (7.4)
**You refer the child to facility with pediatrician/specialist**					
Never	0 (0)	8 (8.1)	1 (1.0)	13 (14.0)	13 (7.4)
Sometimes	3 (10.0)	11 (11.1)	3 (3.0)	22 (23.7)	57 (32.4)
Often	7 (23.3)	17 (17.2)	13 (13.1)	5 (5.4)	15 (8.5)
Always	14 (46.7)	60 (60.6)	25 (25.3)	51 (54.8)	80 (45.5)
Missing	6 (20.0)	3 (3.0)	57 (57.6)	2 (2.2)	11 (6.2)
**Do you wear personal protective equipment before contact with children with TB or presumptive TB?**					
Never	1 (3.3)	31 (31.3)	7 (7.1)	13 (14.0)	15 (8.5)
Sometimes	0 (0)	11 (11.1)	5 (5.1)	13 (14.0)	38 (21.6)
Often	6 (20.0)	10 (10.1)	9 (9.1)	5 (5.4)	17 (9.7)
Always	18 (60.0)	47 (47.5)	30 (30.3)	59 (63.4)	102 (58.0)
Missing	5 (16.7)	0 (0)	48 (48.5)	3 (3.2)	4 (2.3)

**When an adult patient has TB, do you recommend to avoid contact in the household with young children?**					
Never	2 (6.7)	11 (11.1)	6 (6.1)	12 (12.9)	19 (10.8)
Sometimes	1 (3.3)	10 (10.1)	4 (4.0)	18 (19.4)	23 (13.1)
Often	5 (16.7)	13 (13.1)	4 (4.0)	7 (7.5)	13 (7.4)
Always	18 (60.0)	65 (65.7)	43 (43.4)	56 (60.2)	119 (67.6)
Missing	4 (13.3)	0(0.0)	42 (42.4)	0(0.0)	2 (1.1)
**Do you recommend to children with TB or presumptive TB to wear a mask when waiting at the health facility?**					
Never	2 (6.7)	46 (46.5)	6 (6.1)	51 (54.8)	51 (29.0)
Sometimes	0 (0)	13 (13.1)	6 (6.1)	14 (15.1)	31 (17.6)
Often	4 (13.3)	10 (10.1)	4 (4.0)	3 (3.2)	14 (8.0)
Always	20 (66.7)	30 (30.3)	35 (35.4)	25 (26.9)	77 (43.8)
Missing	4 (13.3)	0(0.0)	48 (48.5)	0(0.0)	3 (1.7)
**Do you organize or participate in TB awareness or education sessions in your health facility?**					
Never	5 (16.7)	31 (31.3)	16 (16.2)	11 (11.8)	3 (1.7)
Sometimes	5 (16.7)	31 (31.3)	5 (5.1)	27 (29.0)	55 (31.2)
Often	9 (30.0)	14 (14.1)	18 (18.2)	11 (11.8)	21 (11.9)
Always	6 (20.0)	23 (23.2)	12 (12.1)	43 (46.2)	95 (54.0)
Missing	5 (16.7)	0(0.0)	48 (48.5)	1 (1.1)	2 (1.1)
**Do you do investigation of contacts of a child newly diagnosed with TB?**					
Never	8 (26.7)	36 (36.4)	10 (10.1)	22 (23.7)	16 (9.1)
Sometimes	5 (16.7)	25 (25.3)	1 (1.0)	18 (19.4)	38 (21.6)
Often	8 (26.7)	15 (15.2)	10 (10.1)	9 (9.7)	16 (9.1)
Always	6 (20.0)	23 (23.2)	22 (22.2)	43 (46.2)	100 (56.8)
Missing	3 (10.0)	0 (0.0)	56 (56.6)	1 (1.1)	6 (3.4)
**Do you do child contact tracing for newly diagnosed adult TB cases (index case)?**					
Never	3 (10.0)	40 (40.4)	6 (6.1)	20 (21.5)	10 (5.7)
Sometimes	4 (13.3)	15 (15.2)	5 (5.1)	21 (22.6)	38 (21.6)
Often	7 (23.3)	21 (21.2)	14 (14.1)	11 (11.8)	13 (7.4)
Always	11 (36.7)	23 (23.2)	20 (20.2)	40 (43.0)	107 (60.8)
Missing	5 (16.7)	0(0.0)	54 (54.5)	1 (1.1)	8 (4.5)
**Do you provide TB Preventive Therapy to asymptomatic child contacts of newly diagnosed patients?**					
Never	12 (40.0)	35 (35.4)	11 (11.1)	30 (32.3)	23 (13.1)
Sometimes	4 (13.3)	15 (15.2)	5 (5.1)	27 (29.0)	33 (18.8)

Often	2 (6.7)	12 (12.1)	7 (7.1)	6 (6.5)	19 (10.8)
Always	9 (30.0)	37 (37.4)	14 (14.1)	28 (30.1)	93 (52.8)
Missing	3 (10.0)	0 (0.0)	62 (62.6)	2 (2.2)	8 (4.5)

Supplementary Tables S4.5 & 4.6. Case study around childhood TB management

A child aged 3 years comes in with a persistent cough of 16 days duration, the cough is dry and the child is not short of breath. According to his mother, the child is less playful, looks very tired and has been feeling the same way for the past four weeks

	**Cambodia n (%)**	**Cameroon n (%)**	**Côte d'Ivoire n (%)**	**Sierra Leone n (%)**	**Uganda n (%)**
What likely diagnosis do you suspect?					
HIV	1 (3.3)	0 (0)	5 (5.1)	9 (9.7)	6 (3.4)
Acute respiratory infection/pneumonia	9 (30.0)	42 (42.4)	15 (15.2)	16 (17.2)	17 (9.7)
Asthma	0 (0.0)	1 (1.0)	2 (2.0)	0 (0.0)	6 (3.4)
Tuberculosis	17 (56.7)	56 (56.6)	37 (37.4)	67 (72.0)	147 (83.5)
Missing	3 (10.0)	0 (0.0)	40 (40.4)	1 (1.1)	0 (0.0)
How would you confirm your likely diagnosis?					
Ask for recent contact with TB patient	19 (63.3)	24 (24.2)	22 (22.2)	22 (23.7)	41 (23.3)
Clinical evaluation	1 (3.3)	19 (19.2)	6 (6.1)	16 (17.2)	31 (17.6)
HIV test	0 (0)	1 (1.0)	4 (4.0)	6 (6.5)	5 (2.8)
Xpert test	5 (16.7)	27 (27.3)	6 (6.1)	28 (30.1)	66 (37.5)
Malaria RTD	0 (0.0)	0 (0)	1 (1.0)	1 (1.1)	0 (0)
Tuberculin Skin Test	1 (3.3)	14 (14.1)	5 (5.1)	3 (3.2)	9 (5.1)
X-ray	2 (6.7)	14 (14.1)	13 (13.1)	14 (15.1)	23 (13.1)
**Missing**	2 (6.7)	0 (0.0)	42 (42.4)	3 (3.2)	1 (0.6)

A 6-year old child presents at the health facility after 1 week of antibiotics. He's feeling weak and he has been coughing for 4 weeks now. The mother explains that she came 10 days ago and that a test was done, it was negative. In your register you see that a sputum smear was done and it was negative.

	**Cambodia n (%)**	**Cameroon n (%)**	**Côte d'Ivoire n (%)**	**Sierra Leone n (%)**	**Uganda n (%)**
Do you refer the child for chest-X-Ray?					
Never	4 (13.3)	8 (8.1)	22 (12.5)	19 (20.4)	22 (12.5)
Sometimes	5 (16.7)	8 (8.1)	58 (33.0)	17 (18.3)	58 (33.0)
Often	4 (13.3)	16 (16.2)	18 (10.2)	5 (5.4)	18 (10.2)
Always	12 (40.0)	67 (67.7)	76 (43.2)	50 (53.8)	76 (43.2)
Missing	5 (16.7)	0 (0.0)	2 (1.1)	2 (2.2)	2 (1.1)
Do you send the child home with a new course of antibiotics?					
Never	13 (43.3)	63 (63.6)	77 (43.8)	39 (41.9)	77 (43.8)
Sometimes	3 (10.0)	15 (15.2)	54 (30.7)	33 (35.5)	54 (30.7)
Often	4 (13.3)	12 (12.1)	10 (5.7)	6 (6.5)	10 (5.7)
Always	3 (10.0)	9 (9.1)	32 (18.2)	13 (14.0)	32 (18.2)
Missing	7 (23.3)	0 (0.0)	3 (1.7)	2 (2.2)	3 (1.7)

Do you try to do another TB test/refer for another TB test?					
Never	5 (16.7)	12 (12.1)	15 (8.5)	15 (16.1)	15 (8.5)
Sometimes	5 (16.7)	17 (17.2)	51 (29.0)	24 (25.8)	51 (29.0)
Often	7 (23.3)	14 (14.1)	24 (13.6)	5 (5.4)	24 (13.6)
Always	6 (20.0)	56 (56.6)	82 (46.6)	47 (50.5)	82 (46.6)
Missing	7 (23.3)	0 (0.0)	4 (2.3)	2 (2.2)	4 (2.3)

Supplementary Table S4.7. Practices of childhood TB management according to key HCW characteristics and by country.

	District			Facility level			Position in facility			Contract type			Experience		
	District A	District B	*p value*	DH	PHC	*p value*	Direct TB care	Indirect TB care	*p value*	Non-permanent	Permanent	*p value*	<10 years	>10 years	*p value*
**Diagnose children with TB or presumptive TB few times a week**															
Cambodia	0.0	25.0	0.143	0.0	22.2	0.348	16.7	11.1	0.100	12.5	13.6	0.296	7.1	18.8	0.167
Cameroon	17.4	18.9	0.349	23.3	15.9	**<.001**	17.6	19.4	0.735	20.4	16.0	0.284	18.1	18.8	0.762
Côte d'Ivoire	13.3	16.7	0.922	16.7	13.3	0.633	17.9	11.6	0.418	13.8	17.1	0.949	16.0	11.1	0.405
Sierra Leone	33.3	35.3	0.856	32.1	37.5	0.312	30.2	43.3	0.180	37.5	32.8	0.649	31.7	54.5	0.232
Uganda	18.2	37.5	**<.001**	28.4	27.5	0.572	27.1	28.8	0.798	25.0	28.5	0.815	27.8	28.6	0.716
**Never start children on TB treatment without laboratory confirmation**															
Cambodia	64.3	75.0	0.509	54.5	77.8	0.898	75.0	66.7	0.407	62.5	72.7	0.694	71.4	68.8	0.170
Cameroon	84.8	79.2	0.482	70.0	87.0	0.018	83.8	77.4	0.739	87.8	76.0	0.062	83.1	75.0	0.363
Côte d'Ivoire	37.8	44.4	0.510	35.2	48.9	0.370	48.2	32.6	0.170	34.5	51.2	0.573	42.0	38.9	0.935
Sierra Leone	47.6	66.7	0.242	45.3	75.0	**0.023**	55.6	63.3	0.899	56.2	59.0	0.348	59.8	45.5	0.608
Uganda	38.6	35.2	0.542	48.6	28.4	**0.002**	34.4	40.0	0.692	37.5	36.8	0.931	36.4	42.9	0.706
**Ask for chest X-Ray systematically in all children with presumptive TB**															
Cambodia	0.0	6.2	0.641	9.1	0.0	**0.014**	0.0	5.6	0.811	0.0	4.5	0.531	0.0	6.2	**0.039**
Cameroon	17.4	22.6	0.925	30.0	15.9	0.312	25.0	9.7	0.208	22.4	18.0	0.729	22.9	6.2	0.319
Cote d'Ivoire	4.4	18.5	0.411	11.1	13.3	0.898	17.9	4.7	0.120	6.9	19.5	0.065	14.8	0.0	0.361
Sierra Leone	4.8	11.8	0.905	5.7	12.5	0.211	7.9	10.0	0.920	12.5	6.6	0.738	8.5	9.1	0.069
Uganda	5.7	10.2	0.868	9.5	6.9	0.470	11.5	3.8	0.175	15.6	6.2	**0.053**	7.4	14.3	**0.031**
**Always wear personal protective equipment before contact with children with TB or presumptive TB**															
Cambodia	64.3	56.2	0.201	63.6	55.6	0.276	50.0	66.7	0.201	62.5	59.1	0.515	57.1	62.5	0.897
Cameroon	37.0	56.6	0.209	33.3	53.6	0.085	44.1	54.8	0.240	57.1	38.0	0.077	48.2	43.8	0.605
Côte d'Ivoire	22.2	37.0	0.143	37.0	22.2	**0.043**	32.1	27.9	0.848	24.1	39.0	0.501	27.2	44.4	0.310
Sierra Leone	64.3	62.7	0.301	58.5	70.0	0.683	60.3	70.0	0.627	59.4	65.6	0.779	62.2	72.7	0.422
Uganda	54.5	61.4	0.541	59.5	56.9	0.553	52.1	65.0	0.225	53.1	59.0	0.416	58.0	57.1	0.991
**Always provide TB preventive therapy to asymptomatic child contacts of newly diagnosed patients**															
Cambodia	42.9	18.8	0.212	18.2	38.9	0.139	50.0	16.7	**0.034**	25.0	31.8	1.00	28.6	31.2	0.118

Cameroon	41.3	34.0	0.124	30.0	40.6	0.025	38.2	35.5	0.717	34.7	40.0	0.856	37.3	37.5	0.837
Côte d'Ivoire	11.1	16.7	0.345	11.1	17.8	0.660	21.4	4.7	0.067	8.6	22.0	0.160	13.6	16.7	0.661
Sierra Leone	35.7	25.5	0.172	30.2	30.0	0.706	33.3	23.3	0.625	31.2	29.5	0.405	32.9	9.1	0.269
Uganda	48.9	56.8	0.286	52.7	52.9	0.861	53.1	52.5	0.812	50.0	53.5	0.976	52.5	57.1	0.861
